# Developing updated and new guidance to promote reliable patient identification

**DOI:** 10.1093/jamiaopen/ooaf160

**Published:** 2026-01-06

**Authors:** Emma Weatherford, Anne Grauer, Carina Sirochinsky, I-Fong Lehman, Neha Thummala, Miriam Callahan, Dean F Sittig, Hardeep Singh, Hojjat Salmasian, Madison Jurgens, Jason S Adelman

**Affiliations:** Vagelos College of Physicians and Surgeons, Columbia University, New York, NY 10032, United States; Department of Medicine, Columbia University Irving Medical Center, New York, NY 10032, United States; Center for Patient Safety Science, Columbia University, New York, NY 10032, United States; Vagelos College of Physicians and Surgeons, Columbia University, New York, NY 10032, United States; Center for Patient Safety Science, Columbia University, New York, NY 10032, United States; Center for Patient Safety Science, Columbia University, New York, NY 10032, United States; Center for Patient Safety Science, Columbia University, New York, NY 10032, United States; Department of Surgery, Columbia University Irving Medical Center, New York, NY 10032, United States; Department of Clinical and Health Informatics, McWilliams School of Biomedical Informatics, University of Texas Health Science Center, Houston, TX 77030, United States; Center for Innovations in Quality, Effectiveness and Safety, Michael E. DeBakey Veterans Affairs Medical Center and Baylor College of Medicine, Houston, TX 77030, United States; Division of General Internal Medicine, Harvard Medical School, Boston, MA 02115, United States; Department of Quality and Safety, Brigham and Women’s Hospital, Boston, MA 02115, United States; Augustus C. Long Health Sciences Library, Columbia University Irving Medical Center, New York, NY 10032, United States; Department of Medicine, Columbia University Irving Medical Center, New York, NY 10032, United States; Center for Patient Safety Science, Columbia University, New York, NY 10032, United States; Department of Quality and Patient Safety, NewYork-Presbyterian, New York, NY 10032, United States

**Keywords:** electronic health records, patient identification systems, patient safety, biometric identification, wrong-patient error

## Abstract

**Objectives:**

To describe the process of updating the Patient Identification Safety Assurance Factors for EHR Resilience (SAFER) Guide and to review new practices and refinements to the Guide.

**Materials and Methods:**

We conducted a review of literature on the topic of patient identification in healthcare settings, focusing on papers published after 2016. Titles and abstracts were screened by a team of reviewers, and the full text of retained articles was used to inform the revision.

**Results:**

The updated SAFER Guide strengthens recommendations for displaying patient photographs and using electronic patient identification, including barcoding and radiofrequency identification on patient wristbands. The Guide also recommends the use of biometric identification at registration and point of care. Finally, the updated Guide removes a recommendation to restrict the number of concurrently open patient records permitted in the electronic health record.

**Discussion:**

The revised SAFER Guide includes new recommendations aimed at supporting accurate patient identification at registration, order placement, and the point of care.

**Conclusion:**

The updated Patient Identification SAFER Guide provides evidence-based national recommendations to help reduce patient misidentification.

## Introduction

Accurate patient identification is critical to patient safety, yet identification errors remain widespread.^1^^,^^2^ In 1 case, psychotropic medications were ordered for a neonate after a provider mistook “MC [male child] Jane Doe” for his mother.^3^ In another, a healthy kidney was removed from a man who shared the name of a patient scheduled for nephrectomy.^4^ In a particularly devastating incident, a patient found in cardiac arrest was permitted to die without intervention; subsequent investigation revealed that the “do not resuscitate” order followed by his team was filed for another patient, whose record was inadvertently accessed during the code.^1^ Even when sentinel events like these are avoided, errors in patient identification can harm patients through unnecessary procedures, medication, or testing; delayed care; and compromised confidentiality.

Although electronic health records (EHRs) have provided new tools to prevent wrong-patient errors, they have also created novel opportunities for patient misidentification. Evidence-based patient safety recommendations are needed to guide reliable and accurate patient identification in the EHR. In recent years, many studies have evaluated strategies to prevent patient misidentification. In this case report, we describe the process of revising guidelines for patient identification in the EHR to reflect this new evidence.

## Background

In 2014, the Office of the National Coordinator for Health Information Technology (ONC), now the Assistant Secretary for Technology Policy (ASTP), published the first Safety Assurance Factors for EHR Resilience (SAFER) Guides. The SAFER Guides summarize best practices for EHR safety in several domains, including patient identification. Since 2022, hospitals eligible for reimbursement through the Medicare and Medicaid Promoting Interoperability Program have been required to assess their compliance with the SAFER Guides annually.^5^

The SAFER Guides were updated in 2016.^5^^,^^6^ Since that time, significant social, technological, and regulatory changes have altered the landscape of health information technology. Meanwhile, new research has increased knowledge about patient safety interventions. To bring the recommendations up to date, a revision of all SAFER Guides was undertaken in 2024.^7^ This paper focuses on the revision of the Patient Identification SAFER Guide.

## Methods

To inform the revision process, we undertook a review of the literature on patient identification published since the last update of the SAFER Guides. The review was informed by JBI guidelines for scoping reviews and Cochrane resources for rapid reviews.^8^^,^^9^ However, methods were adjusted to ensure the search strategy remained comprehensive while achieving the goals of this project, which was intended primarily to produce evidence-based guidelines rather than to map existing knowledge on this topic.

Search strategies were iteratively developed using keywords and subject headings extracted from the SAFER recommendations and other literature on this topic.^10^ We developed search strings for 9 themes identified within the 2016 Guide (patient registration; verification of patient identity; organizational monitoring of identification errors; patient lists; check digits; name alerts; deceased patients; restriction of concurrent open charts; and EHR testing, training, and backup) as well as for a broad search for literature relevant to patient identification. On July 12, 2024, literature searches were conducted in PubMed (NCBI), Embase (Elsevier), CINAHL (EBSCO), and Scopus (Elsevier). Results for each theme were saved in an EndNote library.

Because the literature review was intended to update the 2016 guidelines, we retained only literature published after 2016. Search results were screened against additional inclusion criteria by a team of reviewers. Articles were retained if the full text was available in English and if the paper addressed practices intended to promote reliable patient identification in the EHR or described the circumstances of patient misidentification. Most forms of published and grey literature were eligible for inclusion, but unpublished academic literature (eg, preprints) was excluded. During the screening process, recommendations related to the theme of EHR testing, training, and backup were moved to another SAFER Guide; consequently, this theme’s library was excluded from further screening and analysis. The results of the screening process are summarized in Supplementary Appendix A.

## Results

Our search identified 896 unique papers published since 2017. Of these papers, 148 were retained after screening (Supplementary Appendix B). Additional papers were identified through citation tracking.

Based on the results of the literature review, reviewers edited, added, and deleted recommendations from the Guide. In addition, we graded each recommended practice according to a new system developed for the updated SAFER Guides.^7^ Practices were graded as required (mandated by law or regulation), strong (supported by studies showing improvements associated with the practice), medium (supported by common sense, expert opinion, or reports of adverse events in the absence of the practice), or low (commonly used by high-performing health organizations).

The final set of recommended practices is summarized in Table 1. (The full text of the updated Guide is available in Supplementary Appendix C.) Changes from the 2016 Guide are summarized in Table 2. Some recommendations from the 2016 Guide were merged or split, while others were updated with new examples or evidence. Additionally, 3 practices were added to the Guide, while a recommendation from the 2016 Guide was deleted.

**Table 1.** Summary of the 2024 Patient Identification SAFER Guide.

Theme	Recommendation(s)	Strength
Patient registration	An enterprise-wide master patient index that includes patients’ demographic information and medical record number is used to identify patients before importing data (Rec 1.1)	Medium
	Patients are registered in a centralized, common database using standardized procedures (Rec 2.1)	Medium
	The organization has a process to assign temporary, unique patient IDs (which are later merged into permanent IDs) for when the patient registration system is unavailable, or when patients cannot be registered under their legal names (Rec 2.2)	Required
Organizational monitoring	The organization monitors for patient identification errors (Rec 3.1)	Strong
	The organization monitors and rapidly remediates errors that stem from the failure to create, access, and maintain 1 unique medical record for each patient (Rec 3.2)	Strong
Verification of patient identity	Information required to accurately identify the patient is clearly displayed on all portions of the EHR user interface (Rec 1.3)	Strong
	Materials printed from the EHR such as wristbands, labels, and reports include multiple patient identifiers and, in the inpatient setting, an electronic means of verifying patients’ identity (Rec 1.4)	Strong
	The organization uses electronic patient identification such as barcode scanning or radiofrequency identification of patient wristbands to confirm patients’ identity at key points of inpatient care (Rec 2.3)	Strong
	The organization uses biometrics to verify patient identity at registration and at the point of care prior to select patient care practices (Rec 2.4)	Medium
	Patient photographs are collected during patient registration and displayed in multiple places in the EHR to improve patient identification (Rec 2.5)	Strong
Selection from patient lists	To facilitate correct patient identification, clinicians have the ability to create a personalized electronic list of their patients according to several criteria and patient names on adjacent lines of the EHR are displayed in a visually distinct manner (Rec 1.2)	Strong
Check digits	Medical record numbers incorporate a check digit to help prevent data entry errors (Rec 1.5)	Medium
Name alerts	Users are warned when they attempt to create a record for a new patient whose first and last names are the same as another patient, or when a patient search result returns multiple patients with the same or similar names (Rec 1.6)	Medium
Deceased patients	Patients who have died are accurately and clearly identified as deceased (Rec 2.6)	Medium

Abbreviation: EHR, electronic health record.

**Table 2.** Changes to the 2016 SAFER Guide.

Change	2016 practice(s)	2024 practice(s)	Rationale
Addition	N/A	Rec 2.3: The organization uses electronic patient identification such as barcode scanning or radiofrequency identification of patient wristbands to confirm patients’ identity at key points of inpatient care	Electronic patient identification improves compliance with positive patient identification mandates and reduces wrong-patient errors
Addition	N/A	Rec 2.4: The organization uses biometrics to verify patient identity at registration and at the point of care prior to select patient care practices	Biometrics are ubiquitous patient identifiers that improve the accuracy of identification at registration and the point of care
Expansion	Rec 1.4, Examples of potentially useful practices: All computer-generated EHR user interface windows incorporate full name, date of birth, gender, medical record number, location, recent photograph, responsible physician	Rec 2.5: Patient photographs are collected during patient registration and displayed in multiple places in the EHR to improve patient identification	Display of patient photographs in the EHR has been demonstrated to reduce the risk of wrong-patient order errors
Deletion	Rec 2.5: The EHR limits the number of patient records that can be displayed on the same computer at the same time to 1	N/A	An RCT and several quasi-experimental studies found no correlation between the number of concurrently open records permitted and the rate of wrong-patient errors
Merge	Rec 1.2: Clinicians have the ability to create personalized electronic lists of their patients according to several criteria Rec 1.4: Patient names on adjacent lines in the EHR display are visually distinct	Rec 1.2: Clinicians have the ability to create a personalized electronic list of their patients according to several criteria and patient names on adjacent lines of the EHR are displayed in a visually distinct manner	Both recommendations dealt with the design of patient lists and were more effectively discussed in 1 recommendation
Division	Rec 3.1: The organization regularly monitors its patient database for patient identification errors and potential duplicate patients or records	Rec 3.1: The organization monitors for patient identification errors Rec 3.2: The organization monitors and rapidly remediates errors that stem from the failure to create, access, and maintain 1 unique medical record for each patient	This expansive topic was split into recommendations dealing with how an organization monitors errors and how the organization responds once an error is identified
Relocation	Rec 2.2: The user interfaces of the training, test, and read-only backup environments of the EHR are clearly different from the production (ie, “live”) version Rec 2.7: The use of test patients in the production (ie, “live”) environment is carefully monitored. When they do exist, they have unambiguously assigned “test” names and are clearly identifiable as test patients	N/A	These topics were relocated to the new Systems Management SAFER Guide

*Addition*, not previously included in the Guide; *Expansion*, previously included as part of another recommendation; *Deletion*, removed from the updated Guide; *Merge*, combined with another existing recommendation; *Division*, split into multiple recommendations; *Relocation*, moved to another SAFER Guide.

Abbreviations: EHR, electronic health record; N/A, not applicable.

## Discussion

The revised SAFER Guide includes new recommendations aimed at supporting accurate patient identification at registration, order placement, and the point of care. Each of these points presents opportunities for identification errors. At registration, matching algorithms may fail to identify a patient’s existing record, leading to the creation of duplicate charts, or may inaccurately match a patient to another person’s record.^11^^,^^12^ During order placement, providers may select the wrong patient from an electronic list—especially if the list contains patients with similar names—or place an order in the wrong record if interrupted while making treatment decisions.^13–15^ And at the point of care, active verification of multiple patient identifiers is mandated in order to prevent misidentification, but this is an error-prone process and sometimes skipped altogether.^16^ The Guide recommends additional EHR-based practices to address each of these problems.

The updated Guide recommends the use of biometric identifiers at both registration and the point of care. Biometrics—physical characteristics such as fingerprints, vein patterns, and faces—meet many criteria of an ideal identifier. They are specific to an individual, routinely available, and fairly constant over time. They are ubiquitous, possessed by patients regardless of factors such as age and citizenship status. Moreover, they are impossible to steal, leave behind, or give away, and are exceptionally difficult to falsify. Due to these advantages, biometric identification is widely used for identification in contexts from air travel to banking. While usage is more common in the Global South,^17^ healthcare organizations in the United States have also adopted biometrics. Harris Health System in Texas asks patients to “give a low 5” at registration so that their palm vein patterns can be read, while Northwell Health in New York uses facial recognition to simplify check-in before office visits.^18^ At registration, biometrics can be used alongside other data to accurately match patients to their records.^19^ At the point of care, biometrics can be combined with other identifiers for more accurate positive patient identification; for example, medication carts can require a scan of the patient’s palm vein before dispensing medications.^20^ In addition to reducing wrong-patient errors, such a system guarantees that the patient’s identity is confirmed at the bedside, since biometrics can only be scanned in the patient’s presence.

The application of biometrics to patient identification is limited by privacy laws, which sometimes restrict the use of biometric data for identification.^19^^,^^21^ In addition, biometric technology is not equally accessible to all patients: for example, facial recognition software may be less accurate for Black patients.^22^ Such variations in performance not only impede efficient identification but may also be discriminatory if they result in inferior access for some populations.^22^ The SAFER Guide encourages organizations to consider local needs in identifying biometrics that will be widely acceptable and effective.

In addition to biometrics, electronic patient identification (ePID) can improve the accuracy of positive patient identification at the point of care. The most-studied form of ePID is barcoding, in which a barcode linked to identifying information is printed on patient wristbands and scanned before providing care. Barcoding has been shown to improve compliance with positive patient identification requirements and reduce the rate of wrong-patient errors.^23–27^ In 1 analysis, rates of wrong blood in tube errors were more than 3 times lower in facilities using ePID compared with those relying on manual methods.^25^ Another form of ePID, radiofrequency identification (RFID), is not yet widely used for patient identification; however, existing studies have found that RFID shows promise for accurate, efficient ePID.^28^^,^^29^ While the 2016 Guide mentioned barcoding as one way to confirm patient identity, the updated Guide recommends that all organizations use barcoding, RFID, or another form of ePID at key points of care.

Electronic patient identification is not without limitations as a means of ensuring positive patient identification. For example, equipment failures can render an ePID system nonfunctional.^23^^,^^30^ Moreover, barcode-based systems can be bypassed by scanning a copy of the patient’s wristband away from the bedside, thus satisfying the system’s requirements without confirming the patient’s identity at the point of care.^23^^,^^31^ To mitigate these problems, the Guide encourages organizations to conduct performance testing, so that workflow problems can be identified and fixed, and to monitor use of ePID after rollout.

Finally, to prevent errors at order placement, the Guide recommends that a patient photograph be displayed within the EHR. Displaying patient photographs in the EHR has proven to be an effective means of reducing wrong-patient order errors, as photos provide visual cues that help clinicians recognize when they are in the wrong record. In a study conducted in a simulated clinical environment, participants recognized wrong-patient errors faster and at an earlier stage when records included photographs.^14^ Moreover, 2 large quasi-experimental studies found that wrong-patient order errors were significantly less likely when a photograph of the patient was displayed in the EHR.^32^^,^^33^ Based on these data, the updated SAFER Guide recommends that organizations systematically collect patient photographs for display throughout the EHR. This recommendation strengthens existing guidance from the 2016 Guide, which included patient photos in a list of suggested patient identifiers to be displayed in the EHR.

Robust evidence supports patient photos as a tool to prevent identification errors. However, the limitations of this practice are poorly understood. For example, questions remain regarding the correlation between photo quality and prevention of wrong-patient errors. Further study is needed to evaluate questions like this one.

In addition to adding the 3 recommendations reviewed above, we removed from the updated Guide a recommendation that EHRs permit the display of only 1 patient record at a time. When the SAFER Guides were last updated, expert opinion and qualitative research suggested that permitting multiple concurrently open records would increase the risk of wrong-patient errors.^15^^,^^34^^,^^35^ A 2013 study attributed a majority of wrong-patient order errors to interruptions; providers reported that after toggling between records to deal with an interruption, they sometimes remained in the wrong record to place orders.^15^ To prevent this scenario, the 2016 SAFER Guide recommended that providers be permitted to open only 1 record at a time.

However, subsequent studies, including a randomized clinical trial and several quasi-experimental studies, found that restricting concurrently open records did not reduce the rate of wrong-patient errors.^2^^,^^36–38^ Instead, such restrictions appeared to create new safety hazards: to meet the demands of patient care, providers reported devising risky workarounds, such as using a colleague’s log-in credentials or relying on verbal orders.^39^ In addition, restricted systems were perceived as inefficient and unsatisfactory, contributing to frustration with the EHR.^39^^,^^40^ Because restricting the number of open records does not appear to reduce wrong-patient errors, the SAFER Guide no longer recommends a limit on the number of concurrently open records.

## Conclusion

The Patient Identification SAFER Guide provides a roadmap for translating current knowledge about patient identification into practice. The recent revision brings these guidelines up to date with emerging technologies and new evidence. However, the issue of patient identification will continue to evolve in response to novel technologies, updated research, and shifting social norms. Ongoing evaluation is needed to assess emerging practices and to maintain evidence-based guidelines.

## Supplementary Material

ooaf160_Supplementary_Data

## Data Availability

The revised SAFER Guides are available on the ASTP website (https://www.healthit.gov/topic/safety/safer-guides) as well as in Supplementary Appendix C.
