# Supplementary material for: Developing updated and new guidance to promote reliable patient identification
Source: JAMIA Open. 2026 Jan 6;9(1):ooaf160. doi: 10.1093/jamiaopen/ooaf160 (PMC12772641; doi:10.1093/jamiaopen/ooaf160)
Supplement: ooaf160_Supplementary_Data [file ooaf160_supplementary_data.docx]

**APPENDICES**

**Appendix A.** Flowchart of screening process

**Appendix B.** List of unique articles retained after screening (n=148)

**Appendix C.** 2024 PATIENT IDENTIFICATION SAFER GUIDE

**Appendix A.** Flowchart of screening process.


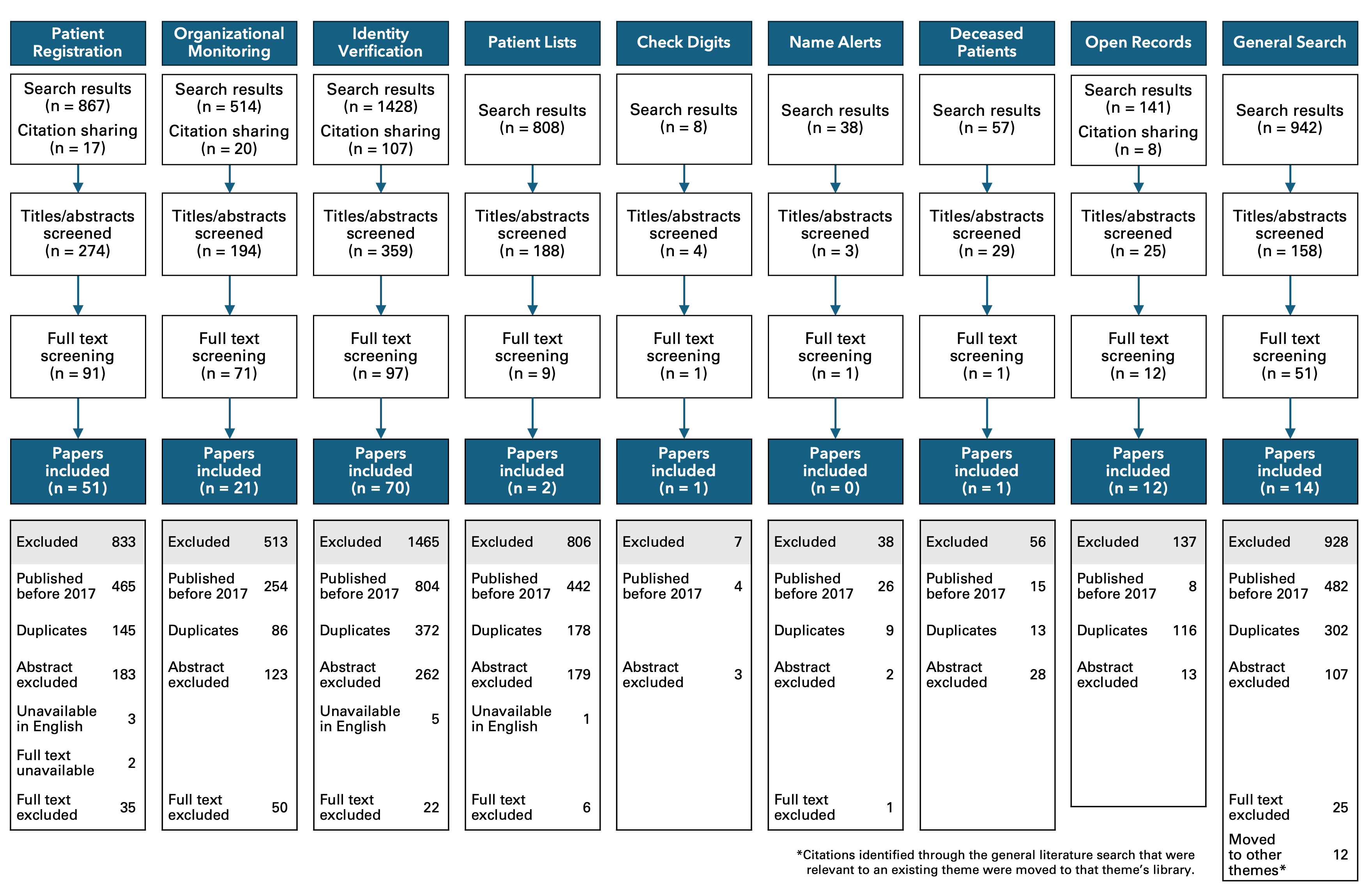
Search results for each of nine themes were screened based on predetermined inclusion criteria, yielding between 0 and 70 retained papers per theme.

**Appendix B. Unique articles retained after screening (n=148)**

**NOTE:** Reference numbers are for count only and do not indicate numeric citations. The difference in number of references in the 2024 SAFER Guide and the tally of reviewed articles here is because not all of the below references were cited in the Guide, and some older citations from the 2016 Guide carried over to the 2024 Guide.

**2024**

1. Rzewnicki D, Kanvinde A, Gillespie S, Orenstein E. Association of patient photographs and reduced retract-and-reorder events. *JAMIA Open* 2024; 7 (3): ooae042
2. Sivo A, Yarbrough K, Weston R. Implementation of a standardized pre-procedure handoff bundle. *J Radiol Nurs* 2024; 43 (2): 128–138
3. Saraswat S, Singh S, Middha P, Thirwani P, Rohilla H. Revolutionizing pandemic healthcare: mask detection and patient face recognition. Proceedings of the 14th International Conference on Cloud Computing, Data Science and Engineering, Confluence 2024 2024; 888–892

**2023**

1. Barboi C, Dixon BE, McFarlane TD, Grannis SJ. Chapter 12 - Client registries: identifying and linking patients. In: Dixon BE (ed) Health Information Exchange (2nd Edition). Academic Press 2023; 275–302
2. Hua Y, Wang L, Nguyen V, et al. A deep learning approach for transgender and gender diverse patient identification in electronic health records. *J Biomed Inform* 2023; 147: 104507
3. Kneifati-Hayek JZ, Applebaum JR, Schechter CB, et al. Effect of restricting electronic health records on clinician efficiency: substudy of a randomized clinical trial. *J Am Med Inform Assoc* 2023; 30 (5): 953–957
4. Myronuk LDS. Effects on wrong-patient errors by limiting access to concurrently open ERH charts: a preliminary systematic mapping and synthesis review. Medrxiv.org preprint 2023
5. Reid DT, Haider M, Lake E, Josyula S, Gosser R, Wu C. Enhancing patient safety by adding patient photos to the electronic medical record of a primary care clinic serving non-English speaking immigrants. *J Gen Intern Med* 2023; 38: S646–S647
6. Suclupe S, Kitchin J, Sivalingam R, McCulloch P. Evaluating patient identification practices during intrahospital transfers: a human factors approach. *J Patient Saf* 2023; 19 (2): 117–127
7. Deng Y, Gleason LP, Culbertson A, et al. Evolving availability and standardization of patient attributes for matching. *Health Aff Sch* 2023; 1 (4): qxad047
8. Omotoye KA, Misra S, Kaushik M, Ogundokun R. Facial liveness detection in biometrics: a multivocal literature review. Information Systems and Management Science Conference Proceedings of 4th International Conference on Information Systems and Management Science (ISMS) 2021 2023; 195–209
9. Nelson W, Khanna N, Ibrahim M, et al. Optimizing patient record linkage in a master patient index using machine learning: algorithm development and validation. *JMIR Form Res* 2023; 7: e44331
10. Al-Eshaq DH, Bradley RT, McBride ERA, Ford JC. Patient and specimen identification in a tertiary care pediatric hospital: barcodes do not scan themselves. *Transfusion* 2023; 63 (7): 1310–1317
11. Chapman S. Patient identification challenges. For the Record (Great Valley Publishing Company, Inc.) 2023; 35 (3): 18–21
12. Alkhaqani AL. Patient identification errors in the hospital setting: a prospective observational study. *Al-Rafidain J Med Sci* 2023; 4: 1–5
13. Van Hal C, Mills JL, Gatmaitan M, Yang G. A patient-centered approach to collecting and displaying patient identifiers. *Stud Health Technol Inform* 2023; 310: 369–373
14. Thriemer N, Emonds J, Knels L, Schlißke S, Polze N, Henschler R. Pretransfusional electronic crossmatch prevents transfusion to the wrong patient. *Transfus Med Hemother* 2023; 50: 17
15. Mutemaringa T, Heekes A, Smith M, Boulle A, Tiffin N. Record linkage for routinely collected health data in an African health information exchange. *Int J Popul Data Sci* 2023; 8 (1): 1771

**2022**

1. Sawa M, Inoue T, Manabe S. Biometric palm vein authentication of psychiatric patients for reducing in-hospital medication errors: a pre-post observational study. *BMJ Open* 2022; 12 (4): e055107
2. Li X, Xu H, Grannis S. The data-adaptive Fellegi-Sunter model for probabilistic record linkage: algorithm development and validation for incorporating missing data and field selection. *J Med Internet Res* 2022; 24 (9): e33775
3. Barro SG, Ugon A, Nana NR, Staccini P. Design and implementation of a unique patient identification model in information systems in Burkina Faso. *Stud Health Technol Inform* 2022; 290: 238–242
4. Grannis SJ, Williams JL, Kasthuri S, Murray M, Xu H. Evaluation of real-world referential and probabilistic patient matching to advance patient identification strategy. *J Am Med Inform Assoc* 2022; 29 (8): 1409–1415
5. Gupta AK, Kasthurirathne SN, Xu H, et al. A framework for a consistent and reproducible evaluation of manual review for patient matching algorithms. *J Am Med Inform Assoc* 2022; 29 (12): 2105–2109
6. Shi Y, Ye C, Wang H, et al. The impact of a closed-loop electronic blood transfusion system on transfusion errors and staff time in a children's hospital. *Transfus Clin Biol* 2022; 29 (3): 250–252
7. Furuta Y, Nakamura Y, Tokida M, et al. Implementation of an electronic identification system in the setting of perioperative autologous cell salvage transfusion: experience at a university hospital. *Transfus Apher Sci* 2022; 61 (1): 103281
8. Rigotti JN, Quraishy NJ, Walker LA. Improvement in transfusion safety via use of an electronic tracking system: Is it enough? *Transfusion* 2022; 62: 245A
9. Steitz BD, Li G, Wright A, Dunworth B, Freundlich RE, Wanderer JP. Non-interruptive clinical decision support to improve perioperative electronic positive patient identification. *J Med Syst* 2022; 46 (3): 15
10. Reuland BD, Redman CT, Kneifati-Hayek JZ, et al. Observation and patients' perceptions of incorporating their photograph into the electronic health record. *J Patient Saf* 2022; 18 (5): 377–381
11. Kulju S, Morrish W, King L, Bender J, Gunnar W. Patient misidentification events in the Veterans Health Administration: a comprehensive review in the context of high-reliability health care. *J Patient Saf* 2022; 18 (1): e290–e296
12. Woodward M, Nayak R, McCulloch P. Radiography as a sociotechnical system – improving patient identification with a multi-level human factors approach. *Safety Science* 2022; 150
13. Alshehri M, Alamri A, Alghamdi M, Nazer R, Kujan O. Smart-card technology for the dental management of medically complex patients. *Healthcare (Basel)* 2022; 10 (11)
14. Campbell A, Ok S, Esguerra J, et al. Using a patient identification checklist: how to make this a never safety event in perioperative services. *J Perianesth Nurs* 2022; 37 (4): e2–e2
15. Nayeri ND, Nadali J, Divani A, Hatefimoadab N. Ways to enhance blood transfusion safety: a systematic review. *Florence Nightingale J Nurs* 2022; 30 (3): 288–300
16. Adair D, Lusk K, Podczervinski R, Smiley C. The year ahead: ONC and AHIMA Project US@ collaboration for improved patient matching. *J AHIMA (Online)* 2022

**2021**

1. Udeh C, Canfield C, Briskin I, Hamilton AC. Association between limiting the number of open records in a tele-critical care setting and retract-reorder errors. *J Am Med Inform Assoc* 2021; 28 (8): 1791–1795
2. Tay KY, Pang YH, Ooi SY, Goh FL. Contactless patient authentication for registration using face recognition technology. *Lecture Notes in Electrical Engineering* 2021; 724: 71–80
3. Hutton K, Ding Q, Wellman G. The effects of bar-coding technology on medication errors: a systematic literature review. *J Patient Saf* 2021; 17 (3): e192–e206
4. Meren ÜH, Waterson J. Evaluating an automated compounding workflow software for safety and efficiency: implementation study. *JMIR Hum Factors* 2021; 8 (4): e29180
5. Jaafa NK, Mokaya B, Savai SM, Yeung A, Siika AM, Were M. Implementation of fingerprint technology for unique patient matching and identification at an HIV care and treatment facility in western Kenya: cross-sectional study. *J Med Internet Res* 2021; 23 (12): e28958
6. De Rezende H, Melleiro MM, Marques PAO, Barker TH. Interventions to reduce patient identification errors in the hospital setting: a systematic review. *Open Nurs J* 2021; 15 (1): 109–121
7. Morishita J, Ueda Y. New solutions for automated image recognition and identification: challenges to radiologic technology and forensic pathology. *Radiol Phys Technol* 2021; 14 (2): 123–133
8. Crew D, Houser SH. Overcoming challenges of merging multiple patient identification and matching systems: a case study. *Perspect Health Inf Manag* 2021; 18 (Winter): 1n
9. Dennison D. Patient Identity Management Maturity Model (PIM3) for imaging information technology systems. *J Digit Imaging* 2021; 34 (2): 473–482
10. Clinician.com newsletter. Patient photos in electronic health record cut wrong order entries by 35% (executive summary). Healthcare Risk Management 2021; 43 (4): 1–2
11. Khunlertkit A, Dorissaint L, Chen A, Paine L, Pronovost PJ. Reducing and sustaining duplicate medical record creation by usability testing and system redesign. *J Patient Saf* 2021; 17 (7): e665–e671
12. Davies J, Milser E, Tuckley V, et al. SHOT 2019 key recommendations survey. *Transfus Med* 2021; 31 (SUPPL 1): 41–42
13. Bolton-Maggs PHB. Strategies to eliminate errors in blood transfusions. *Vox Sang* 2021; 116 (SUPPL 1): 12
14. Abraham P, Augey L, Duclos A, Michel P. Descriptive analysis of patient misidentification from incident report system data in a large academic hospital federation. *J Patient Saf* 2021; 17 (7): e615–e621

**2020**

1. Besagar S, Robles PL, Rojas C, Applebaum JR, Adelman JS, Goffman D. Acceptability of using newborns' given names at birth: survey in postpartum and antepartum units. *Obstet Gynecol* 2020; 135: 156S–157S
2. Ross MK, Sanz J, Tep B, Follett R, Soohoo SL, Bell DS. Accuracy of an electronic health record patient linkage module evaluated between neighboring academic health care centers. *Appl Clin Inform* 2020; 11 (5): 725–732
3. Gibney B, Ryan JW, MacMahon PJ, O'Connor GS, Bolster F. Assessment of RAdiopaque Patient IDentification Stickers (RAPIDS) for patient-scan correlation in a mass casualty incident. *Emerg Radiol* 2020; 27 (3): 293–301
4. Salmasian H, Blanchfield BB, Joyce K, et al. Association of display of patient photographs in the electronic health record with wrong-patient order entry errors. *JAMA Netw Open* 2020; 3 (11): e2019652
5. Moscovitch B, Halamka JD, Grannis S. Better patient identification could help fight the coronavirus. *NPJ Digit Med* 2020; 3 (1)
6. Sohn JW, Kim H, Park SB, et al. Clinical study of using biometrics to identify patient and procedure. *Front Oncol* 2020; 10: 586232
7. Stanuch M, Wodzinski M, Skalski A. Contact-free multispectral identity verification system using palm veins and deep neural network. *Sensors (Basel)* 2020; 20 (19)
8. Sittig DF, Wright A, Coiera E, et al. Current challenges in health information technology–related patient safety. *Health Informatics J* 2020; 26 (1): 181–189
9. Redfield C, Tlimat A, Halpern Y, et al. Derivation and validation of a machine learning record linkage algorithm between emergency medical services and the emergency department. *J Am Med Inform Assoc* 2020; 27 (1): 147–153
10. Pfeifer E, Lozovatsky M, Abraham J, Kannampallil T. Effect of an alternative newborn naming strategy on wrong-patient errors: a quasi-experimental study. *Appl Clin Inform* 2020; 11 (2): 235–241
11. Barakat S, Franklin BD. An evaluation of the impact of barcode patient and medication scanning on nursing workflow at a UK teaching hospital. *Pharmacy (Basel)* 2020; 8 (3)
12. Anne N, Dunbar MD, Abuna F, et al. Feasibility and acceptability of an iris biometric system for unique patient identification in routine HIV services in Kenya. *Int J Med Inform* 2020; 133: 104006
13. Kim TC, JL Howe JL, ES Franklin ES, et al. Health information technology-related wrong-patient errors: context is critical. *Patient Saf (Harrisbg)* 2020; 2 (4): 40–57
14. Staples S, O'Callaghan C, Pavord S, Staves J, Murphy MF. How to verify patient identity and blood product compatibility using an electronic bedside transfusion system. *Transfusion* 2020; 60 (9): 2153–2155
15. Vanneman MW, Balakrishna A, Lang AL, et al. Improving transfusion safety in the operating room with a barcode scanning system designed specifically for the surgical environment and existing electronic medical record systems: an interrupted time series analysis. *Anesth Analg* 2020; 131 (4): 1217–1227
16. Tridandapani S, Bhatti P, Krupinski E, Safdar N, Wick C. Initial experience with patient visible light images obtained simultaneously with portable radiographs. *AJR Am J Roentgenol* 2020; 214 (1): 68–71
17. Canfield C, Udeh C, Blonsky H, Hamilton AC, Fertel BS. Limiting the number of open charts does not impact wrong patient order entry in the emergency department. *J Am Coll Emerg Physicians Open* 2020; 1 (5): 1071–1077
18. Goffman D, Kern-Goldberger A, Fernandes Y, Salmasian H, Applebaum JR, Adelman JS. Mother-baby errors: a potential unintended consequence of distinct newborn naming conventions. *Obstet Gynecol* 2020; 135: 163S
19. Riplinger L, Piera-Jiménez J, Dooling JP. Patient identification techniques - approaches, implications, and findings. *Yearb Med Inform* 2020; 29 (1): 81–86
20. Ampamya S, Kitayimbwa JM, Were MC. Performance of an open source facial recognition system for unique patient matching in a resource-limited setting. *Int J Med Inform* 2020; 141: 104180
21. Cohen JK. Playing the match game: biometrics, algorithms help boost hospitals' patient-matching rates. Modern Healthcare (Crain Communications, Inc) 2020; 50 (23): 16–16
22. Etter L, Simukanga A, Qin W, et al. Project SEARCH (Scanning EARs for Child Health): validating an ear biometric tool for patient identification in Zambia. *Gates Open Res* 2020; 4: 168
23. Clinician.com newsletter. Registration mistakes can harm patients clinically. Hospital Access Management 2020; 39 (11): 1–2
24. Qian S, Munyisia E, Reid D, Hailey D, Pados J, Yu P. Trend in data errors after the implementation of an electronic medical record system: a longitudinal study in an Australian regional drug and alcohol service. *Int J Med Inform* 2020; 144
25. T Shinzaki S. Use case of palm vein authentication. In: Uhl A, Busch C, Marcel S, Veldhuis R (eds). Handbook of Vascular Biometrics. Advances in Computer Vision and Pattern Recognition. Springer, Cham 2020; 145–158
26. Thomas JJ, Yaster M, Guffey P. The use of patient digital facial images to confirm patient identity in a children's hospital's anesthesia information management system. *Jt Comm J Qual Patient Saf* 2020; 46 (2): 118–121
27. Fortman E, Hettinger AZ, Howe JL, et al. Varying rates of patient identity verification when using computerized provider order entry. *J Am Med Inform Assoc* 2020; 27 (6): 924–928
28. Akers C, Pickles L, Haberfield A, Davis A. Wrong blood in tube (WBIT): Pre and post introduction of electronic medical record. *Vox Sang* 2020; 115 (SUPPL 1): 110
29. Kern-Goldberger AR, Adelman J, Applebaum JR, Manzano W, Goffman D. Wrong-patient ordering errors in peripartum mother-newborn pairs: a unique patient-safety challenge in obstetrics. *Obstet Gynecol* 2020; 136 (1): 161–166

**2019**

1. Blanchfield BB, Salmaisian H, Landman A. Adding patient photos to the electronic health record to improve patient identification and reduce wrong patient order errors. *Ann Emerg Med* 2019; 74 (4): S22–S23
2. Callum J, Etchells E, Shojania K. Addressing the identity crisis in healthcare: positive patient identification technology reduces wrong patient events. *Transfusion* 2019; 59 (3): 899–902
3. Ueda Y, Morishita J, Hongyo T. Biological fingerprint using scout computed tomographic images for positive patient identification. *Med Phys* 2019; 46 (10): 4600–4609
4. Katsanis SH, Huang E, Young A, et al. Caring for trafficked and unidentified patients in the EHR shadows: shining a light by sharing the data. *PLoS One* 2019; 14 (3): e0213766
5. Southern WN, Applebaum JR, Salmasian H, et al. Clinician experience of electronic health record configurations displaying 1 vs 4 records at a time. *JAMA Intern Med* 2019; 179 (12): 1723–1725
6. Scariati P, Knapp H, Gray S. A comparison of one- and four-open-chart access: no change in computerized provider order entry error rates. *Appl Clin Inform* 2019; 10 (5): 804–809
7. Frangella J, Cassarino M, Plazzotta F, Gassino F, Otero C, Luna D. Designed strategies and adaptation of a master patient index for transgender patients in a tertiary care hospital. *Stud Health Technol Inform* 2019; 264: 1698–1699
8. Nagels J, Wu S, Gorokhova V. Deterministic vs. probabilistic: best practices for patient matching based on a comparison of two implementations. *J Digit Imaging* 2019; 32 (6): 919–924
9. MacMaster HW, Gonzalez S, Maruoka A, et al. Development and implementation of a subcutaneous insulin pen label bar code scanning protocol to prevent wrong-patient insulin pen errors. *Jt Comm J Qual Saf* 2019; 45 (5): 380–386
10. Chapman S. Diversity key to patient matching. *For the Record (Great Valley Publishing Company, Inc.)* 2019; 31 (1): 18–21
11. Etter LP, Ragan EJ, Campion R, Martinez D, Gill CJ. Ear biometrics for patient identification in global health: a field study to test the effectiveness of an image stabilization device in improving identification accuracy. *BMC Med Inform Decis Mak* 2019; 19 (1): 114
12. Adelman JS, Applebaum JR, Schechter CB, et al. Effect of restriction of the number of concurrently open records in an electronic health record on wrong-patient order errors: a randomized clinical trial. *JAMA* 2019; 321 (18): 1780–1787
13. Kaufman RM, Dinh A, Cohn CS, et al. Electronic patient identification for sample labeling reduces wrong blood in tube errors. *Transfusion* 2019; 59 (3): 972–980
14. Grannis SJ, Xu H, Vest JR, et al. Evaluating the effect of data standardization and validation on patient matching accuracy. *J Am Med Inform Assoc* 2019; 26 (5): 447–456
15. Jeon B, Jeong B, Jee S, et al. A facial recognition mobile app for patient safety and biometric identification: design, development, and validation. *JMIR Mhealth Uhealth* 2019; 7 (4): e11472
16. Chou SS, Chen YJ, Shen YT, Yen HF, Kuo SC. Implementation and effectiveness of a bar code-based transfusion management system for transfusion safety in a tertiary hospital: retrospective quality improvement study. *JMIR Med Inform* 2019; 7 (3): e14192
17. Huang Y. Improve clinic blood component supply and assure blood transfusion safety by enhancing a blood transfusion database platform. *Vox Sang* 2019; 114 (SUPPL 2): 34
18. Oza S, Wing K, Sesay AA, et al. Improving health information systems during an emergency: lessons and recommendations from an Ebola treatment centre in Sierra Leone. *BMC Med Inform Decis Mak* 2019; 19 (1): 100
19. Cidon D. Industry perspectives. Data standardization can improve patient matching. *For the Record (Great Valley Publishing Company, Inc.)* 2019; 31 (6): 28–29
20. De Rezende HA, Melleiro MM, Shimoda GT. Interventions to reduce patient identification errors in the hospital setting: a systematic review protocol. *JBI Database System Rev Implement Rep* 2019; 17 (1): 37–42
21. Blonksy H, Shirley R, Milinovich A, et al. Limiting the number of open charts in the electronic medical record does not decrease order entry errors in cardiovascular inpatient and intensive care units. *Circulation* 2019; 140
22. Canfield C, Hamilton AC, Udeh C, et al. Limiting the number of open charts in the electronic medical record does not decrease order errors in the emergency department. *Ann Emerg Med* 2019; 74 (4): S21
23. Su CT. Limiting the number of open records in an electronic health record. *JAMA* 2019; 322 (13): 1313–1314
24. Adelman JS, Applebaum JR, Southern WN. Limiting the number of open records in an electronic health record-reply. *JAMA* 2019; 322 (13): 1314–1315
25. Kasthurirathne SN, Grannis SJ. Machine learning approaches to identify nicknames from a statewide health information exchange. *AMIA Jt Summits Transl Sci Proc* 2019; 2019: 639–647
26. Khalil EGA, Osman ASA. A novel method for patients identification in emergency cases using RFID based radio technology. *Int J Adv Comput Sci Appl* 2019; 10 (12): 468–471
27. Tridandapani S, Bhatti P, Wick C. Patient photographs: privacy versus protection. *AJR Am J Roentgenol* 2019; 212 (2): 320–322
28. Vijenthira S, Armali C, Downie H, et al. Registration errors among patients receiving blood transfusions: a national analysis from 2008-2017. *Blood* 2019; 134: 3689
29. Wachter RM, Murray SG, Adler-Milstein J. Restricting the number of open patient records in the electronic health record: Is the record half open or half closed? *JAMA* 2019; 321 (18): 1771–1773
30. Adelman JS, Applebaum JR, Southern WN, et al. Risk of wrong-patient orders among multiple vs singleton births in the neonatal intensive care units of 2 integrated health care systems. *JAMA Pediatr* 2019; 173 (10): 979–985
31. La Madrid D, Barriga M, Shiguihara P. Technological model of facial recognition for the identification of patients in the health sector. In: Iano Y, Arthur R, Saotome O, Vieira Estrela V, Loschi H (eds). Proceedings of the 4th Brazilian Technology Symposium (BTSym 2018). Smart Innovation, Systems and Technologies. Springer, Cham 2019; 140: 595–603
32. Cohen R, Ning S, Yan MTS, Callum J. Transfusion safety: the nature and outcomes of errors in patient registration. *Transfus Med Reviews* 2019; 33 (2): 78–83
33. Ferguson C, Hickman L, Macbean C, Jackson D. The wicked problem of patient misidentification: How could the technological revolution help address patient safety? *J Clin Nurs* 2019; 28 (13-14): 2365–2368
34. Hensley NB, Koch CG, Pronovost PJ, et al. Wrong-patient blood transfusion error: leveraging technology to overcome human error in intraoperative blood component administration. *Jt Comm J Qual Patient Saf* 2019; 45 (3): 190–198

**2018**

1. Kaufman RM, Yazer MH, Dinh A, et al. Collecting pretransfusion samples using electronic patient identification reduces wrong blood in tube errors. *Transfusion* 2018; 58: 18A
2. Rudin RS, Hillestad R, Ridgely MS, Qureshi N, Davis II JS, Fischer SH. Defining and evaluating patient-empowered approaches to improving record matching. Santa Monica, CA: RAND Corporation 2018
3. Kannampallil TG, Manning JD, Chestek DW, et al. Effect of number of open charts on intercepted wrong-patient medication orders in an emergency department. *J Am Med Inform Assoc* 2018; 25 (6): 739–743
4. Saathoff AM, MacDonald R, Krenzischek E. Effectiveness of specimen collection technology in the reduction of collection turnaround time and mislabeled specimens in emergency, medical-surgical, critical care, and maternal child health departments. *Comput Inform Nurs* 2018; 36 (3): 133–139
5. Arndt RZ. Fail-safe patient matching remains just out of reach. Modern Healthcare (Crain Communications, Inc) 2018; 48 (29): 0022–0022
6. McGann HL, Reese EM, Fontaine MJ, Grewal AS, Hebrank A. Implementation of blood component transfusion using electronic positive patient identification within the intra-operative suites. *Transfusion* 2018; 58: 155A–156A
7. Larsen E, Fong A, Wernz C, Ratwani RM. Implications of electronic health record downtime: an analysis of patient safety event reports. *J Am Med Inform Assoc* 2018; 25 (2): 187–191
8. Taieb-Maimon M, Plaisant C, Hettinger AZ, Shneiderman B. Increasing recognition of wrong-patient errors through improved interface design of a computerized provider order entry system. *Int J Hum Comput Interact* 2018; 34 (5): 383–398
9. Clinician.com newsletter. Joint Commission Advisory addresses ensuring accurate patient identification. Same-Day Surgery 2018
10. Soe ZN, Ali S. Local experience of 'Wrong blood in the tube', rejected transfusion samples; Hull and East Yorkshire hospitals NHS trust. *Br J Haematol* 2018; 181: 154
11. Brown B, Balatsoukas P, Williams R, Sperrin M, Buchan I. Multi-method laboratory user evaluation of an actionable clinical performance information system: implications for usability and patient safety. *J Biomed Inform* 2018; 77: 62–80
12. Hcpro.com newsletter. Patient identification errors: solve mistakes to reduce denials and risk. HIM Briefings 2018; 33 (9): 1–5
13. Elkins S. Patient matching: Are we any closer to a solution? For The Record Magazine 2018; 30 (9): 18
14. Ning S, Yan MTS, Downie H, Callum J. What's in a name? Patient registration errors and their threat to transfusion safety. *Transfusion* 2018; 58 (12): 3035–3036

**2017**

1. Gomes KM, Riggs SL. Analyzing visual search techniques using eye tracking for a computerized provider order entry (CPOE) task. Proceedings of the Human Factors and Ergonomics Society Annual Meeting 2017; 61 (1): 691–695
2. Adelman JS, Aschner JL, Schechter CB, et al. Babyboy/Babygirl: A national survey on the use of temporary, nondistinct naming conventions for newborns in neonatal intensive care units. *Clinical Pediatrics* 2017; 56 (12): 1157–1159
3. Paaske S, Bauer A, Moser T, Seckman C. The benefits and barriers to RFID technology in healthcare. *Online J Nurs Inform* 2017; 21 (2): 10–1
4. Culbertson A, Goel S, Madden MB, et al. The building blocks of interoperability. A multisite analysis of patient demographic attributes available for matching. *Appl Clin Inform* 2017; 8 (2): 322–336
5. Albornoz MA, Márquez S, Rubin L, Luna D. Design of a mobile application for transfusion medicine. *Stud Health Technol Inform* 2017; 245: 994–998
6. Manning JD, Chestek DW, Bunney EB, Kannampallil T, Galanter W. Do the number of charts open at one time affect wrong-patient medication error rates? *Acad Emerg Med* 2017; 24: S74
7. Lim SC, Koh AJH, Poon EWH. Electronic display board in operating theatres for easy patient identification. *BMJ Open Qual* 2017; 6 (2): e000021
8. Daus M, Maydana T, Lede DR, Luna D. Enhancing children's safety by barcoding implementation at breast milk feeding. *Stud Health Technol Inform* 2017; 245: 49–53
9. Adelman JS, Aschner JL, Schechter CB, et al. Evaluating serial strategies for preventing wrong-patient orders in the NICU. *Pediatrics* 2017; 139 (5)
10. Kantartjis M, Melanson SEF, Petrides AK, et al. Increased patient satisfaction and a reduction in pre-analytical errors following implementation of an electronic specimen collection module in outpatient phlebotomy. *Lab Med* 2017; 48 (3): 282–289
11. Lippi G, Mattiuzzi C, Bovo C, Favaloro EJ. Managing the patient identification crisis in healthcare and laboratory medicine. *Clin Biochem* 2017; 50 (11-Oct): 562–567
12. Millan A, Mingo A, Gonzalez MI, Mena A, Benitez JP. A new RFID transfusion safety system. *Transfusion* 2017; 57: 167A–168A
13. Lippi G, Chiozza L, Mattiuzzi C, Plebani M. Patient and sample identification. Out of the maze? *J Med Biochem* 2017; 36 (2): 107–112
14. Addison J, Ball J, Mistry H, Poles D, Bolton-Maggs P. Preventing wrong component transfusion-importance of the final administration check. *Vox Sang* 2017; 112: 146
15. Hagger-Johnson G, Harron K, Goldstein H, Aldridge R, Gilbert R. Probabilistic linkage to enhance deterministic algorithms and reduce data linkage errors in hospital administrative data. *J Innov Health Inform* 2017; 24 (2): 891
16. Clinician.com newsletter. Report finds thousands of patient ID errors. Hospital Access Management 2017; 36 (1)
17. Waruhari P, Babic A, Nderu L, Were MC. A review of current patient matching techniques. *Stud Health Technol Inform* 2017; 238: 205–208
18. Licher J, Köhn J, Loutfi-Krauß B, Scherf C, Ramm U. Risk management in radiotherapy - patient identification and patient verification. *Biomedizinische Technik* 2017; 62: S252
19. Yigzaw KY, Michalas A, Bellika JG. Secure and scalable deduplication of horizontally partitioned health data for privacy-preserving distributed statistical computation. *BMC Med Inform Decis Mak* 2017; 17 (1): 1

**2016**

1. Basavatia A, Fret J, Lukaj A, et al. Right care for the right patient each and every time. *Cureus* 2016; 8 (2): e492
2. Le RD, Melanson SEF, Petrides AK, et al. Significant reduction in preanalytical errors for nonphlebotomy blood draws after implementation of a novel integrated specimen collection module. *Am J Clin Pathol* 2016; 146 (4): 456–461
3. McCoy AB, Wright A, Kahn MG, Shapiro JS, Bernstam EV, Sittig DF. Matching identifiers in electronic health records: implications for duplicate records and patient safety. *BMJ Qual Saf* 2013; 22 (3): 219
4. Morrison AP, Tanasijevic MJ, Goonan EM, et al. Reduction in specimen labeling errors after implementation of a positive patient identification system in phlebotomy. *Am J Clin Pathol* 2010; 133 (6): 870–877

**APPENDIX C:** 2024 PATIENT IDENTIFICATION SAFER GUIDE

**Introduction**

The *Patient Identification SAFER Guide* identifies recommended safety practices associated with the reliable identification of patients in the EHR. Accurate patient identification ensures that the information displayed and entered into the EHR is associated with the correct person. Processes related to patient identification are complex and require careful planning and attention to avoid errors. In the EHR-enabled healthcare environment, providers rely on technology to help support and manage these complex identification processes. Technology configurations alone cannot ensure accurate patient identification.^1^ Staff also must be supported with adequate training and reliable procedures.

This Patient Identification self-assessment can help identify and evaluate where breakdowns related to patient identification occur in the healthcare setting. It focuses on processes within organizations related to the creation of new patient records, patient registration, retrieval of information on previously registered patients, and other types of patient identification activities. The updated recommended practices can help prevent or detect and mitigate problems caused by duplicate records, patient mix-ups, and “comingled” (or “overlay”) records.^2-11^

This guide is meant to support and enable patient matching technology and capabilities, focusing on best practices for improving data accuracy, which is the first step to ensuring accurate patient matching. Although patient matching between organizations is not the focus of this guide, examples herein demonstrate their potential value and typical scenarios in which they are used.

The recommended practices in this Patient Identification SAFER Guide provide support for many, varied patient matching technologies, as well as alternatives and best practices on specific patient attributes for patient matching, which are likely to change over time. New evidence on the importance of incorporating appropriate interventions such as the display of patient photographs, barcoding, and palm scanning are discussed. Other research herein highlights emerging issues related to EHR systems, internal workflow processes, and their potential interactions and impacts.

Completing the self-assessment in the Patient Identification SAFER Guide requires the engagement of people both within and outside the organization (e.g., EHR technology vendors). Because this guide is designed to help organizations prioritize EHR-related safety concerns, clinician leadership in the organization should be engaged in assessing whether and how any particular recommended practice affects the organization’s ability to deliver safe, high-quality care. Collaboration between clinicians and staff members while completing the self-assessment in this guide will enable an accurate snapshot of the organization’s patient identification status (in terms of safety), and even more importantly, should lead to a consensus about the organization’s future path to optimize EHR-related safety and quality: setting priorities among the recommended practices not yet addressed, ensuring a plan is in place to maintain recommended practices already in place, dedicating the required resources to make necessary improvements, and working together to prevent and mitigate the highest priority patient identification-related safety risks introduced by the EHR.

**Table of Contents**

**Safe Health IT**

Rec 1.1: An Enterprise-wide Master Patient Index (EMPI) is used

Rec 1.2: Personalized patient lists

Rec 1.3: Identifiers on user interfaces are clearly displayed

Rec 1.4: Identifiers on printed materials are clearly displayed

Rec 1.5: Check digits

Rec 1.6: Name alert

**Using Health IT Safely**

Rec 2.1: Standardized registration

Rec 2.2: Temporary identifiers

Rec 2.3: Barcoding and RFID

Rec 2.4: Biometrics

Rec 2.5: Patient photos

Rec 2.6: Deceased patients

**Monitoring Safety**

Rec 3.1: Monitoring of poor compliance of patient identification and wrong patient errors

Rec 3.2: Monitoring failures to create, access, and maintain one unique medical record for each patient

**Recommended Practice 1.1**

An enterprise-wide master patient index (EMPI) is used to identify patients before importing data. The EMPI includes patients’ demographic information and medical record number (MRN) (or multiple numbers if used by different parts of the same organization, along with primary number/key) .^12^

**Rationale for Practice or Risk Assessment**

When patients are not matched accurately to their existing records, their health data can be fragmented across duplicate records or commingled with another patient’s data, leading to patient harm.^13,14^ The occurrence of duplicate records and overlays can be reduced by using an EMPI to identify patients with existing records.^12^ An EMPI also facilitates record deduplication following mergers between healthcare organizations.^15^

**Strength of Recommendation:** Medium

**Suggested Sources of Input**

1. Health IT support staff
2. Registration staff
3. Clinical support staff

**Implementation Guidance**

- The EMPI assigns each patient a unique identifier that is different from the patient’s MRN.
- Registration staff are trained to use the EMPI to look for an existing record before creating a new record.
- Organizational policies address how to use the EMPI to ensure correct patient identification of information from external sources (e.g., external labs, pharmacies, healthcare providers).
- Records with a high degree of similarity that fail to match due to missing demographic data are flagged for manual review.^16,17^
- When a new patient record is created, the registrar is prompted to consider potential matches in the existing database.
- The organization has policies and procedures to prevent creation of duplicate records or overlays. Usability testing is conducted to identify opportunities for improvement.^18^ The organization reviews its EMPI-related policies and procedures at least annually, updating as new recommended practices are defined.
- The EMPI employs a probabilistic matching algorithm that uses patients' first and last names, date of birth, sex, and other attributes (e.g., middle name, zip code, telephone number, last four digits of the Social Security number).^16,18^ Manual adjustment or machine learning are used to tailor the algorithm for greater accuracy within an organization’s context.^18-20^

**Recommended Practice 1.2**

To facilitate correct patient identification, clinicians can create personalized electronic lists of their patients according to several criteria (e.g., user, location, time, service),^21^ and patient names on adjacent lines of the EHR are displayed in a visually distinct manner.^2,21,22^

**Rationale for Practice or Risk Assessment**

Wrong patient selection errors often go unrecognized by clinicians.^23^ Selecting a patient from a shorter list of relevant patients and keeping patient names visually distinct in the EHR reduce the risk of unintentionally selecting the wrong patient.^21-24^

**Strength of Recommendation:** Strong

**Suggested Sources of Input**

1. EHR developer
2. Health IT support staff

**Implementation Guidance**

- Patient lists can be automatically generated in several formats to provide information relevant to clinical or administrative needs: person-specific (e.g., all patients for whom a clinician is responsible), location-specific (e.g., all patients in a particular nursing unit or clinic), time-specific (e.g., all patients on today's schedule), and service- or clinician-specific (e.g., all patients being cared for by a particular specialty, service, or clinician).^2,22^
- Clinicians can view, create, modify, and delete patient lists for their own clinical purposes.^21^
- Patient lists are sorted in a clinically relevant order by default (e.g., by room number, appointment time), rather than alphabetically, to reduce the chance of identical, lookalike, or sound-alike names appearing close together.^2^
- Two or more unique identifiers are included for each patient on the list (e.g., name, date of birth, medical record number, sex/gender).^2,25^
- The patient’s full name is displayed including when preferred name also is displayed.^26^
- Patient list font size and spacing are optimized to reduce the chance of inadvertently selecting the wrong record.^22^
- A patient’s name is highlighted (e.g., by a distinct color, bold or italic font) when their record is selected on a patient list.^22,23^
- On all patient lists containing two or more patients with identical, lookalike, or sound-alike surnames, the names in common are displayed in a visually distinct manner (e.g., bold, italics, different color).^2,22^

**Recommended Practice 1.3**

Information required to accurately identify the patient is clearly displayed on all portions of the EHR user interface.^2^

**Rationale for Practice or Risk Assessment**

Providing medical services to the wrong patient is a frequent, preventable source of patient harm.^24,27^ To reduce the risk of wrong-patient errors, steps should be taken to ensure that the person using an EHR to care for a patient is addressing the intended patient. Patient names alone are not sufficient for identification, as evidenced by scenarios of mislabeled laboratory samples^28^ and the significant proportion of wrong-patient events and close calls where two identifiers were not used.^29^

**Strength of Recommendation:** Strong

**Suggested Sources of Input**

1. EHR developer
2. Health IT support staff

**Implementation Guidance**

- All computer-generated EHR user interface displays incorporate the following information to facilitate patient identification, with appropriate exceptions for individuals for whom such information could create other risks (e.g., survivors of domestic violence): ^2,30,31^
- Full legal name (Last name, first name, middle initial)
- Preferred name, if different from legal name
- Date of birth (with calculated age)
- Legal sex – required for insurance and claims processing
- Gender identity
- Medical record number
- In-patient location (home address or ZIP code for outpatients)
- Recent photograph (**see Rec 2.5**)
- Responsible physician, if applicable
- Patient identifiers in the EHR should be displayed in a manner that promotes identity verification (e.g., using large font sizes, distinct colors, minimal visual clutter, and consistent location across various EHR screens).^31,32^ This information is best displayed on the top-left of the screen, which receives more attention from users.^33^

**Recommended Practice 1.4**

Materials printed from the EHR such as wristbands, labels, and reports include multiple patient identifiers and in the inpatient setting, an electronic means of verifying patients’ identity (e.g., a 1- or 2-dimensional barcode/QR code).

**Rationale for Practice or Risk Assessment**

Materials printed from the EHR must contain multiple patient identifiers (e.g. name, date of birth, medical record number), so that patient’s identity can be verified when the material is distributed to the patient (e.g., postoperative care instructions) or when the material itself is used for verifying patient identity (e.g., wristbands).^34^ Verifying identity solely by confirming a patient’s name and date of birth is subject to human error .^11,35,36^ By incorporating barcodes into the EHR and patient care workflows, identity can be confirmed quickly and reliably by scanning.^35,37^

**Strength of Evidence:** Strong

**Suggested Sources of Input**

1. EHR developer
2. Health IT support staff

**Implementation Guidance**

- All patient-specific materials printed from the EHR include the patient’s full legal name and date of birth alongside a barcode to assist with patient identification.^36,38^
- At time of registration/check-in, patients are issued a wristband including their name, date of birth, and a barcode.^36,38^
- Patient identity is verified at the time of medication administration, specimen collection, procedure performance, and other key moments in patient care by verbally confirming name and date of birth as well as scanning their wristband barcode.^37,38^
- Organizational policies and workflows incorporate use of the EHR to ensure correct patient identification, such as the use of barcodes to verify patient identity at key points in the care delivery process (**see Rec 2.3**).^39,40^

**Recommended Practice 1.5**

Medical record numbers incorporate a check digit to help prevent data entry errors.

**Rationale for Practice or Risk Assessment**

Check digits, an extra number automatically calculated and added to a sequence of numbers to help detect errors, have been incorporated into barcoding programs to improve patient and medication safety^3^ and into personal identification numbers for national registries that are often used by research communities.^41^ Use of check digits contributes to high-quality data collection, mitigates patient ID number mix-ups, can help reduce data entry errors and long-term system errors, assists in the assignment of patient ID numbers to avoid sequential assignments, and reduce errors in critical scenarios where errors are known to increase.^42^

**Strength of Recommendation:** Medium

**Suggested Sources of Input**

1. EHR developer
2. Health IT support staff

**Implementation Guidance**

- To minimize human-generated number insertion, deletion, substitution, or transposition errors or their effects, check digits are utilized to optimize processes for correct patient identification.
- One example of a check digit program is the “Verhoeff algorithm”, which works with strings of decimal digits of any length and detects all single-digit errors and all transposition errors involving two adjacent digits.^43^
- Check digit programs are used in systems that generate pseudo-identifiers for patients whose data are used for research, to reduce data entry errors.^4^

**Recommended Practice 1.6**

Users are warned when they attempt to create a record for a new patient whose first and last names are the same as another patient, or when a patient search result returns multiple patients with the same or similar names.^2^

**Rationale for Practice or Risk Assessment**

Using automated EHR processes to prevent duplicate records can prevent unintentional human errors that could lead to patient harm.^18^ Patients with similar names are at a higher risk for wrong-patient errors.^5^

**Strength of Recommendation:** Medium

**Suggested Sources of Input**

1. EHR developer
2. Health IT support staff

**Implementation Guidance**

- During the creation of a new patient record, a phonetic algorithm such as Soundex^44^ is used to check for patients with similar sounding names in the system and display an alert or warning if one exists.
- When looking up a patient, if the results list returns multiple patients with similar demographic data, the names are displayed in a visually distinct manner.
- The system monitors for similar names, name variants (e.g., Robert, Rob, Bob, Robbie), or changed last names (e.g., marriage, divorce, adoption), when other demographics match.
- An alert provides additional demographic information context for the existing patient to help the user confirm or rule out that it is the same patient.
- Organizations implement an ID reentry intervention and/or a distinct naming intervention to reduce wrong-patient errors in the nursery or NICU, where sets of twins, triplets, and higher-order multiples are prevalent.^5^
- Name alerts in combination with other interventions (e.g., blood type testing) prevent patient record confusion in critical areas such as blood transfusions.^45^

**Recommended Practice 2.1**

Patients are registered in a centralized, common database using standardized procedures.^17,46^

**Rationale for Practice or Risk Assessment**

Standardized entry of full demographic data into a common database at registration improves the accuracy of patient matching and prevents the creation of duplicate charts.^17,46^

**Strength of Recommendation:** Medium

**Suggested Sources of Input**

1. Registration staff
2. Clinicians, support staff, and/or clinical administration
3. Health IT support staff
4. EHR developer

**Implementation Guidance**

- Organizational policy establishes standardized registration procedures involving the EHR and a common database to serve as the “source of truth” on whether a record already exists for a person who presents for services.^6^
- Registration clerks are trained in consistent patient entry practices across portals of entry (e.g., ED, inpatient, clinic, phone, internet). Entry of demographic data is standardized using national or international guidelines when possible for full name,^7^ address,^47^ telephone number,^48^ and sex, and gender identity.^8^
- Patients are asked to provide their full legal names at registration. If possible, legal name is confirmed with government-issued identification.^17^ Preferred names, nicknames, and aliases are recorded in a separate field from legal name**.**^7,49^
- Organizations should determine the minimum set of demographic data required for reliable patient identification and interoperability in their context.^17^ This might include the patient’s first, middle, and last name(s), suffix, previous name(s), date of birth, sex, and current and previous addresses and phone numbers.^9^
- A multiple birth indicator is used when registering pediatric multiple birth patients (twins, triplets, etc.) to prevent subsequent merging of charts based on the similarity of demographic information.^17^
- The organization requires a picture ID to verify the identity of new patients, with appropriate alternatives for minors and others who do not have an official photo ID.^50,51^
- Photo ID or biometrics (e.g., palm vein scanning, fingerprinting, facial recognition) are used to confirm the identity of returning patients.^10,50,52-55^
- Returning patients are asked to verify the accuracy of their demographic data.^17^

**Recommended Practice 2.2**

The organization has a process to assign temporary, unique patient IDs (which are later merged into permanent IDs) for when the patient registration system is unavailable, or when patients cannot be registered under their legal names.^34,56,57^

**Rationale for Practice or Risk Assessment**

In some cases, patients cannot be registered under their legal names. This may occur when typical registration procedures cannot be followed because the patient registration system is unavailable or overwhelmed by a large number of incoming patients.^58,59^ This can also occur when the patient’s identity is unknown (e.g., a trauma victim), when the patient has not been formally named (i.e., a newborn), or when the patient’s safety or privacy could be compromised by use of their legal name (e.g., a public figure or hospital employee). In these circumstances, patients must be assigned a temporary ID, which will later be merged with a permanent ID to avoid maintaining duplicate records.

**Strength of Evidence:** Required

**Suggested Sources of Input**

1. EHR developer
2. Health IT support staff
3. Registration staff
4. Clinicians and clinical support staff

**Implementation Guidance**

- The organization uses a distinctive naming convention for newborns.^34^
  - A temporary ID in the style of Janesboy Smith or BoyJane Smith may be used, with the addition of letters or numbers to distinguish multiple births.^60,61^
  - Alternatively, the child’s given name or a pseudonym may be used.^62^
- Patients whose identities cannot be determined at admission are given IDs that are recognizable as temporary, easily distinguishable by look and sound from other temporary IDs (both in full and in any truncated form used by the organization), and not perceived as dehumanizing or offensive by patients.^56,63-66^
- The organization has a process for providing pseudonyms to patients who wish to disguise their identities for reasons of safety or privacy.
- A process is in place to provide unique temporary IDs to patients when the patient registration system is unavailable or overwhelmed.^57^
- Any downstream use of a temporary ID within a facility, or in transfers between facilities, is tracked and corrected in all electronic systems, including at transfer facilities.^57^
- A process exists to safely merge temporary IDs with permanent ones. If merging occurs during an episode of clinical care, the patient’s name is updated in all systems, and safeguards are in place to prevent confusion about the patient’s identity.^63^
- The organization monitors resolution of temporary IDs.

**Recommended Practice 2.3**

The organization uses electronic patient identification such as barcode scanning or radio-frequency identification of patients’ wristbands to confirm patients’ identity at key points of inpatient care.^67-69^

**Rationale for Practice or Risk Assessment**

To prevent wrong-patient errors, providers should confirm patients’ identity using two identifiers such as name and date of birth at key points of the care process (e.g., prior to procedures and surgeries, vital sign recording, medication administration, specimen collection, and blood transfusion administration).^2,25^ However, manual patient identification is prone to error,^11^ and electronic patient identification – scanning a barcode or using radio-frequency identification (RFID) on a patient’s wristband to confirm the patient’s identity – improves compliance with patient identification and reduces wrong-patient errors.^37,67-69^

**Strength of Evidence:** Strong

**Suggested Sources of Input**

1. Clinicians, support staff, and clinical administration
2. Health IT support staff
3. EHR developer

**Implementation Guidance**

- A patient’s wristband with a patient identification barcode or radiofrequency identification (RFID) is scanned to electronically confirm the patient’s identity prior to procedures and surgeries, vital sign recordings, medication administration, specimen collections, blood transfusion administrations, and at other key points of patient care.
- The EHR prompts providers to use electronic patient identification for patient identity verification.^70^
- Patients, or their healthcare proxy (e.g., for infants, or adults with diminished mental capacity), are asked to confirm their identity verbally in combination with electronic patient identification.^71^
- Patients are informed about the purpose of electronic patient identification and are encouraged to remind providers to use this process.^72^
- Electronic patient identification undergoes performance testing before rollout to identify technical issues, workflow problems, and other barriers to implementation.^73,74^
- The organization maintains a backup manual system for positive patient identification in case of equipment failure, EHR downtime, or other technical difficulties.^57,73^
- Policies, workflows and processes are implemented that aim to optimize electronic patient identification practices and prevent workarounds.^35^
- Reports are created to measure compliance with electronic patient identification practices and performance improvement projects are used to improve compliance.

**R****ecommended Practice 2.4**

The organization uses biometrics to verify patient identity at registration and prior to providing certain types of care.

**Rationale for Practice or Risk Assessment**:

Biometric attributes such as faces, fingerprints, and vein patterns are specific, ubiquitous, and relatively unchanging.^75^ Unlike other patient identifiers, these attributes cannot be stolen, traded, or left behind, and they are difficult to falsify. These factors make biometrics a promising option for confirming patients’ identities, especially at times of high-risk clinical care such as prior to radiation therapy treatments. However, the benefits of using biometrics must be balanced against concerns about privacy and bias.^76,77^

**Strength of Recommendation:** Medium

**Suggested Sources of Input**

1. EHR developer
2. Health IT support staff

**Implementation Guidance**

- Biometric attributes are selected for patient identification with consideration for factors such as privacy, impact on workflow, infection risk (e.g., fomite transmission), feasibility in a given context, and accessibility and acceptability to an organization’s patient population.^53,77-80^
- Patients are given the opportunity to offer informed consent for the collection of biometrics or to opt out.^79^
- When possible, biometrics are gathered from new patients at the time of registration.^50^
- Biometric identification is used as part of patient identification at the point of care,^81^ especially at times of high-risk clinical care such as prior to radiation therapy treatments.
- Biometrics are used in combination with other identifiers to match patients to their existing records.^76^
- The organization has a process to handle a mismatch between a patient’s stated identity and the identity associated with the patient’s biometric data in an existing record. A patient may present under a false name for diverse reasons – for example, to avoid retaliation from a trafficker or to engage in medical identity fraud^54,82^ – which require different responses from the organization.
- Policies, workflows, and processes are implemented that aim to optimize biometric identification practices and prevent workarounds.
- Reports are created to measure compliance with biometric identification practices and performance improvement projects are used to improve compliance.

**Recommended Practice 2.5**

Patient photographs are collected during patient registration and displayed in multiple places in the EHR to improve patient identification.^83^

**Rationale for Practice or Risk Assessment**

The display of color patient photographs in the main banner of an EHR, in patient lists, and in other areas of the EHR, when utilized either on desktop computers or mobile devices, is an effective, non-interruptive method to improve patient identification and reduce wrong patient errors.^10,30,32,83-85^

**Strength of Evidence:** Strong

**Suggested Sources of Input**

1. EHR developer
2. Registration Staff

**Implementation Guidance**

- The organization collects a color photograph of every patient older than three months of age at the time of patient registration, admission to the hospital, or any time staff believe a change in appearance warrants updating the photograph.^10,83,86^
- Patient photographs are displayed in all screens and functions of the EHR supported by the vendor, including patient banners, patient lists, patient scheduling, patient search, and secure messaging.
- Patient photographs are displayed in the EHR in all devices supported by the vendor including desktop computers and mobile devices.
- Policies and practices provide guidance for capturing patient photographs, including when and how to capture them, and describing the optimal patient photo (e.g., the patient’s face is centered and greater than 50% of the image). These practices are sensitive to patient cultural and religious practices with regard to face and head coverings.
- Reports are utilized to monitor the compliance of capturing patient photographs, and performance improvement projects are utilized to improve compliance.
- When patient photographs are not supported by the vendor or not available, other functions are used to improve patient identification such as patient identification alerts or “re-entering” patient identifiers (e.g., initials, name) before signing orders.^5,27^

**Recommended Practice 2.6**

Patients who have died are accurately and clearly identified as deceased.

**Rationale for Practice or Risk Assessment**

Selection of a deceased patient record may lead to a wrong-patient error, yet clear flags identifying that patients have deceased are often missing in EHRs. Clinicians should be able to easily identify that patients they have selected are deceased.^87,88^

**Strength of Recommendation:** Medium

**Suggested Sources of Input**

1. EHR developer
2. Health IT support staff

**Implementation Guidance**

- - - - The EHR should clearly identify which patients are deceased (e.g., through a different background color for the deceased patient header in the EHR or a pop-up alert when opening the record). Care should be taken to avoid using ambiguous, culturally, or religiously insensitive icons.
      - There is a mechanism to verify the death status or indicate that death is unverified (e.g., when the death data is obtained through external data sources).^89,90^
      - Linkage or probabilistic matching algorithms help confirm or supply missing data,^87,88^ and may cross-check EHRs with government data or national registries.^88,91^
      - Accurate death status along with mechanisms to prevent entering billing adjustments as patient visits, removing recurring radiation visits from deceased patient charts, and differentiating classification of post-mortem medical activities such as autopsy procedures and organ donation could greatly reduce instances of apparent post-death health encounters.^87^

**Recommended Practice 3.1**

The organization monitors for patient identification errors.^12,92^

**Rationale for Practice or Risk Assessment**

Patient identification errors are never events that lead to adverse outcomes including death, and should be identified and acted upon as soon as possible.^7^

**Strength of Recommendation:** Strong

**Suggested Sources of Input**

1. EHR developer
2. Health IT support staff

**Implementation Guidance**

- Electronic patient identification practices (e.g. barcoding, biometrics) and internal voluntary reporting error databases are monitored, and performance improvement initiatives are initiated when poor compliance or patient identification hazards are identified.
- The organization has processes to monitor for common scenarios related to wrong patient identification (e.g., changes in patient blood type over time) and to implement corrective actions as needed.^93^
- The NQF-endorsed "retract–and–reorder" (RAR) algorithm is used to measure the rate of wrong patient ordering errors, and corrective actions are implemented as needed.^27,94^

**Recommended Practice 3.2**

The organization monitors and rapidly remediates errors that stem from the failure to create, access, and maintain one unique medical record for each patient (i.e., duplicates, overlays, and overlaps).^6,7^

**Rationale for Practice or Risk Assessment**

Several different process error scenarios have been identified that result in the failure to correctly produce one unique medical record for each patient.^7^ A **duplicate** record is a redundant record created when two or more medical record numbers are created for the same person; an **overlay** occurs when the incorrect patient is registered, admitted, or documented in another patient’s record; and an **overlap** occurs when there is more than one unique patient identifier for the same person across two or more facilities in the enterprise and usually arises after institutional merging.^15^ To minimize patient safety issues, patient misidentification errors, and billing and coding errors,^20,95-97^ organizations must implement strategies to prevent duplicates, overlays, and overlaps, and to correct patient’s records by de-duplicating, disentangling, or merging records when these errors are identified.

**Strength of Recommendation:** Strong

**Suggested Sources of Input**

1. EHR developer
2. Health IT support staff

**Implementation Guidance**

- The organization has a stringent daily process for working with the matching error queue and remediating errors identified to facilitate better patient matching.^7,20,96^
- The organization monitors its duplicate, overlay and overlap error rates, benchmarks them to internal rates quarterly, and ensures that those rates remain at or below industry standards.^7,12,94^
- Once identified, duplicate, overlaid, and overlapped records are immediately remediated.^18,88,98,99^
- In the event that a large number of duplicates are identified, such as during a health system merger, the organization immediately flags those charts as being of concern and creates a time-bound plan for resolving the issues.
- Once identified, duplicate, overlaid, and overlapped records are reviewed to identify any clinical care provided since the creation of the anomalous record situation. Responsible clinicians are notified of the issue so that appropriate patient care interventions can be performed. In addition, the organization should have a policy and procedure describing how these charts should be notated in the event a future medicolegal issue arises.
- The organization/EHR uses algorithms for patient matching that yield the lowest rates of false positives and false negatives to prevent errors from occurring. Machine learning, deep learning, pattern-recognition, natural language processing, and referential matching models^19,20,96,100^ perform better than traditional probabilistic, rules-based, and deterministic algorithms^101,102^ and should be incorporated into patient matching pipelines.

**References**

1. Kowalczyk L. Brigham and Women’s Hospital video uses slapstick to promote patient safety. Boston.Com. 2013. <https://www.boston.com/uncategorized/noprimarytagmatch/2013/03/07/brigham-and-womens-hospital-video-uses-slapstick-to-promote-patient-safety/>. Accessed July 23, 2024.

2. Lowry SZ, Quinn MT, Ramaiah M, et al. Technical evaluation, testing, and validation of the usability of electronic health records. National Institute of Standards and Technology. 2012.

3. Neuenschwander M, Cohen MR, Vaida AJ, Patchett JA, Kelly J, Trohimovich B. Practical guide to bar coding for patient medication safety. *Am J Health Syst Pharm.* 2003;60(8):768-779. <https://pubmed.ncbi.nlm.nih.gov/12749163/>. 10.1093/ajhp/60.8.768; PMID 12749163.

4. Olden M, Holle R, Heid IM, Stark K. Idgenerator: Unique identifier generator for epidemiologic or clinical studies. *BMC Med Res Methodol.* 2016;16:120. <https://pubmed.ncbi.nlm.nih.gov/27628043/>. 10.1186/s12874-016-0222-3; PMID 27628043; PMC5024489.

5. Adelman JS, Aschner JL, Schechter CB, et al. Evaluating serial strategies for preventing wrong-patient orders in the NICU. *Pediatrics.* 2017;139(5). <https://pubmed.ncbi.nlm.nih.gov/28557730/>. 10.1542/peds.2016-2863; PMID 28557730.

6. McCoy AB, Wright A, Kahn MG, Shapiro JS, Bernstam EV, Sittig DF. Matching identifiers in electronic health records: Implications for duplicate records and patient safety. *BMJ Qual Saf.* 2013;22(3):219-224. <https://pubmed.ncbi.nlm.nih.gov/23362505/>. 10.1136/bmjqs-2012-001419; PMID 23362505.

7. American Health Information Management Association (AHIMA). Recommended data elements for capture in the master patient index (MPI). 2021. <https://ahima.org/media/mezosx50/2022-naming-policy-v3-1-21-22.pdf>. Accessed July 29, 2024.

8. Office of the National Coordinator for Health Information Technology (ONC). Sex at birth, sexual orientation and gender identity. 2024. <https://www.healthit.gov/isp/section/sex-birth-sexual-orientation-and-gender-identity>. Accessed July 23, 2024.

9. Office of the National Coordinator for Health Information Technology (ONC). Connecting health and care for the nation: A shared nationwide interoperability roadmap. 2015. <https://www.healthit.gov/sites/default/files/hie-interoperability/nationwide-interoperability-roadmap-final-version-1.0.pdf>. Accessed August 1, 2024.

10. Riplinger L, Piera-Jiménez J, Dooling JP. Patient identification techniques - approaches, implications, and findings. *Yearb Med Inform.* 2020;29(1):81-86. <https://pubmed.ncbi.nlm.nih.gov/32823300/>. 10.1055/s-0040-1701984; PMID 32823300; PMC7442501.

11. Henneman PL, Fisher DL, Henneman EA, Pham TA, Campbell MM, Nathanson BH. Patient identification errors are common in a simulated setting. *Ann Emerg Med.* 2010;55(6):503-509. <https://pubmed.ncbi.nlm.nih.gov/20031263/>. 10.1016/j.annemergmed.2009.11.017; PMID 20031263.

12. Dooling JA, Durkin S, Fernandes L, et al. Managing the integrity of patient identity in health information exchange (updated). *J AHIMA.* 2014;85(5):60-65. <https://pubmed.ncbi.nlm.nih.gov/24938040/>. PMID 24938040.

13. Dennison D. Patient identity management maturity model (PIM3) for imaging information technology systems. *J Digit Imaging.* 2021;34(2):473-482. <https://pubmed.ncbi.nlm.nih.gov/33796987/>. 10.1007/s10278-021-00429-2; PMID 33796987; PMC8289952.

14. Joffe E, Bearden CF, Byrne MJ, Bernstam EV. Duplicate patient records--implication for missed laboratory results. *AMIA Annu Symp Proc.* 2012;2012:1269-1275. <https://pubmed.ncbi.nlm.nih.gov/23304405/>. PMID 23304405; PMC3540536.

15. Crew D, Houser SH. Overcoming challenges of merging multiple patient identification and matching systems: A case study. *Perspect Health Inf Manag.* 2021;18(Winter):1n. <https://pubmed.ncbi.nlm.nih.gov/33633524/>. PMID 33633524; PMC7883361.

16. Just BH, Marc D, Munns M, Sandefer R. Why patient matching is a challenge: Research on master patient index (MPI) data discrepancies in key identifying fields. *Perspect Health Inf Manag.* 2016;13(Spring):1e. <https://pubmed.ncbi.nlm.nih.gov/27134610/>. PMID 27134610; PMC4832129.

17. Heflin E, He S, Isbell K, et al. A framework for cross-organizational patient identity management. 2018. <https://sequoiaproject.org/wp-content/uploads/2018/06/The-Sequoia-Project-Framework-for-Patient-Identity-Management-v31.pdf>. Accessed July 23, 2024.

18. Khunlertkit A, Dorissaint L, Chen A, Paine L, Pronovost PJ. Reducing and sustaining duplicate medical record creation by usability testing and system redesign. *J Patient Saf.* 2021;17(7):e665-e671. <https://pubmed.ncbi.nlm.nih.gov/29076957/>. 10.1097/PTS.0000000000000434; PMID 29076957.

19. Nelson W, Khanna N, Ibrahim M, et al. Optimizing patient record linkage in a master patient index using machine learning: Algorithm development and validation. *JMIR Form Res.* 2023;7:e44331. <https://pubmed.ncbi.nlm.nih.gov/37384382/>. 10.2196/44331; PMID 37384382; PMC10365597.

20. Redfield C, Tlimat A, Halpern Y, et al. Derivation and validation of a machine learning record linkage algorithm between emergency medical services and the emergency department. *J Am Med Inform Assoc.* 2020;27(1):147-153. <https://pubmed.ncbi.nlm.nih.gov/31605488/>. 10.1093/jamia/ocz176; PMID 31605488; PMC7647245.

21. Brown B, Balatsoukas P, Williams R, Sperrin M, Buchan I. Multi-method laboratory user evaluation of an actionable clinical performance information system: Implications for usability and patient safety. *J Biomed Inform.* 2018;77:62-80. <https://pubmed.ncbi.nlm.nih.gov/29146562> /. 10.1016/j.jbi.2017.11.008; PMID 29146562 PMC5766660.

22. Shin GW, Lee Y, Park T, et al. Investigation of usability problems of electronic medical record systems in the emergency department. *Work.* 2022;72(1):221-238. <https://pubmed.ncbi.nlm.nih.gov/34120924/>. 10.3233/WOR-205262; PMID 34120924.

23. Taieb-Maimon M, Plaisant C, Hettinger AZ, Shneiderman B. Increasing recognition of wrong-patient errors through improved interface design of a computerized provider order entry system. *Int J Hum Comput Interact.* 2018;34(5):383-398. 10.1080/10447318.2017.1349249.

24. Mardon R, Olinger L, Szekendi M, Williams T, Sparnon E, Zimmer K. Health information technology adverse event reporting: Analysis of two databases. 2014. <https://www.healthit.gov/sites/default/files/Health_IT_PSO_Analysis_Final_Report_11-25-14.pdf>. Accessed August 1, 2024.

25. The Joint Commission. 2024 Hospital National Patient Safety Goals. 2024. <https://www.jointcommission.org/standards/national-patient-safety-goals/hospital-national-patient-safety-goals/>. Accessed July 29, 2024.

26. Sopan A, Plaisant C, Powsner S, Shneiderman B. Reducing wrong patient selection errors: Exploring the design space of user interface techniques. *AMIA Annu Symp Proc.* 2014;2014:1056-1065. <https://pubmed.ncbi.nlm.nih.gov/25954415/>. PMID 25954415; PMC4420010.

27. Adelman JS, Kalkut GE, Schechter CB, et al. Understanding and preventing wrong-patient electronic orders: A randomized controlled trial. *J Am Med Inform Assoc.* 2013;20(2):305-310. <https://pubmed.ncbi.nlm.nih.gov/22753810/>. 10.1136/amiajnl-2012-001055; PMID 22753810; PMC3638184.

28. Hawker CD, McCarthy W, Cleveland D, Messinger BL. Invention and validation of an automated camera system that uses optical character recognition to identify patient name mislabeled samples. *Clin Chem.* 2014;60(3):463-470. <https://pubmed.ncbi.nlm.nih.gov/24366726/>. 10.1373/clinchem.2013.215434; PMID 24366726.

29. Kulju S, Morrish W, King L, Bender J, Gunnar W. Patient misidentification events in the veterans health administration: A comprehensive review in the context of high-reliability health care. *J Patient Saf.* 2022;18(1):e290-e296. <https://pubmed.ncbi.nlm.nih.gov/32925569/>. 10.1097/pts.0000000000000767; PMID 32925569.

30. Van Hal C, Mills JL, Gatmaitan M, Gong Y. A patient-centered approach to collecting and displaying patient identifiers. *Stud Health Technol Inform.* 2024;310:369-373. <https://pubmed.ncbi.nlm.nih.gov/38269827/>. 10.3233/shti230989; PMID 38269827.

31. Gomes KM, Riggs SL. Analyzing visual search techniques using eye tracking for a computerized provider order entry (CPOE) task. *Proceedings of the Human Factors and Ergonomics Society.* 2017;2017-October:691-695. 10.1177/1541931213601659.

32. Fortman E, Hettinger AZ, Howe JL, et al. Varying rates of patient identity verification when using computerized provider order entry. *J Am Med Inform Assoc.* 2020;27(6):924-928. <https://pubmed.ncbi.nlm.nih.gov/32377679/>. 10.1093/jamia/ocaa047; PMID 32377679; PMC7647277.

33. Segel E, Heer J. Narrative visualization: Telling stories with data. *IEEE Transactions on Visualization and Computer Graphics.* 2010;16(6):1139-1148. 10.1109/TVCG.2010.179.

34. The Joint Commission. National Patient Safety Goals effective January 2024. 2024;2024(July 30). <https://www.jointcommission.org/standards/national-patient-safety-goals/>. Accessed July 31, 2024.

35. Barakat S, Franklin BD. An evaluation of the impact of barcode patient and medication scanning on nursing workflow at a UK teaching hospital. *Pharmacy (Basel).* 2020;8(3). <https://pubmed.ncbi.nlm.nih.gov/32824909/>. 10.3390/pharmacy8030148; PMID 32824909; PMC7560167.

36. De Rezende HA, Melleiro MM, Marques PAO, Barker TH. Interventions to reduce patient identification errors in the hospital setting: A systematic review. *Open Nurs J.* 2021;15:109-121. 10.2174/1874434602115010109.

37. Vanneman MW, Balakrishna A, Lang AL, et al. Improving transfusion safety in the operating room with a barcode scanning system designed specifically for the surgical environment and existing electronic medical record systems: An interrupted time series analysis. *Anesth Analg.* 2020;131(4):1217-1227. <https://pubmed.ncbi.nlm.nih.gov/32925343/>. 10.1213/ane.0000000000005084; PMID 32925343.

38. Saathoff AM, MacDonald R, Krenzischek E. Effectiveness of specimen collection technology in the reduction of collection turnaround time and mislabeled specimens in emergency, medical-surgical, critical care, and maternal child health departments. *Comput Inform Nurs.* 2018;36(3):133-139. <https://pubmed.ncbi.nlm.nih.gov/29120913/>. 10.1097/cin.0000000000000402; PMID 29120913.

39. Ning HC, Lin CN, Chiu DT, et al. Reduction in hospital-wide clinical laboratory specimen identification errors following process interventions: A 10-year retrospective observational study. *PLoS One.* 2016;11(8):e0160821. <https://pubmed.ncbi.nlm.nih.gov/27494020/>. 10.1371/journal.pone.0160821; PMID 27494020; PMC4975414.

40. Dhatt GS, Damir HA, Matarelli S, Sankaranarayanan K, James DM. Patient safety: Patient identification wristband errors. *Clin Chem Lab Med.* 2011;49(5):927-929. <https://pubmed.ncbi.nlm.nih.gov/21288177/>. 10.1515/cclm.2011.129; PMID 21288177.

41. Sund R, Gissler M. *Use of health registers.* New York, NY: Springer New York; 2019.

42. Oza S, Wing K, Sesay AA, et al. Improving health information systems during an emergency: Lessons and recommendations from an ebola treatment centre in Sierra Leone. *BMC Med Inform Decis Mak.* 2019;19(1):100. <https://pubmed.ncbi.nlm.nih.gov/31133075/>. 10.1186/s12911-019-0817-9; PMID 31133075; PMC6537453.

43. Chu M, Kang G, Ryu KH. An improved check digit-based participant identification system for human biorepositories. 2023 Asia Pacific Signal and Information Processing Association Annual Summit and Conference, APSIPA ASC 2023. 2023:1614-1621. <https://www.scopus.com/inward/record.uri?eid=2-s2.0-85180010293&doi=10.1109%2fAPSIPAASC58517.2023.10317197&partnerID=40&md5=90c35b152da43b2c21b9c7fd2cef6c90>.

44. The U.S. National Archives and Records Administration. Soundex System | The Soundex Indexing System. Updated Jan 9, 2024. <https://www.archives.gov/research/census/soundex>. Accessed August 6, 2024.

45. Ferrera-Tourenc V, Lassale B, Chiaroni J, Dettori I. Unreliable patient identification warrants ABO typing at admission to check existing records before transfusion. *Transfus Clin Biol.* 2015;22(2):66-70. <https://pubmed.ncbi.nlm.nih.gov/25936944/>. 10.1016/j.tracli.2015.03.004; PMID 25936944.

46. Grannis SJ, Xu H, Vest JR, et al. Evaluating the effect of data standardization and validation on patient matching accuracy. *J Am Med Inform Assoc.* 2019;26(5):447-456. <https://pubmed.ncbi.nlm.nih.gov/30848796/>. 10.1093/jamia/ocy191; PMID 30848796; PMC7787357.

47. Project US@. Project US@: Technical specifications for patient addresses, domestic and military. 2022:65. <https://asapnet.org/wp-content/uploads/2022/03/Project_US_FINAL_Technical_Specification_Version_1.0.pdf>. Accessed August 1, 2024.

48. International Telecommunication Union. ITU Recommendation E.123: Notation for national and international telephone numbers, e-mail addresses and web addresses. 2001. <https://www.itu.int/itu-t/recommendations/rec.aspx?rec=5341>. Accessed July 24, 2024.

49. Frangella J, Cassarino M, Plazzotta F, Gassino F, Otero C, Luna D. Designed strategies and adaptation of a master patient index for transgender patients in a tertiary care hospital. *Stud Health Technol Inform.* 2019;264:1698-1699. <https://pubmed.ncbi.nlm.nih.gov/31438299/>. 10.3233/SHTI190603; PMID 31438299.

50. Abel L, Buegel RA, Dooling JA, et al. Best practices for patient matching at patient registration. *J AHIMA.* 2016;87(10):74-81.

51. LeBrón AMW, Cowan K, Lopez WD, Novak NL, Ibarra-Frayre M, Delva J. It works, but for whom? Examining racial bias in carding experiences and acceptance of a county identification card. *Health Equity.* 2018;2(1):239-249. <https://pubmed.ncbi.nlm.nih.gov/30283873/>. 10.1089/heq.2018.0022; PMID 30283873; PMC6167006.

52. Stanuch M, Wodzinski M, Skalski A. Contact-free multispectral identity verification system using palm veins and deep neural network. *Sensors (Basel).* 2020;20(19). <https://pubmed.ncbi.nlm.nih.gov/33036259/>. 10.3390/s20195695; PMID 33036259; PMC7582870.

53. Waruhari P, Babic A, Nderu L, Were MC. A review of current patient matching techniques. *Stud Health Technol Inform.* 2017;238:205-208. <https://pubmed.ncbi.nlm.nih.gov/28679924/>. PMID 28679924.

54. Katsanis SH, Huang E, Young A, et al. Caring for trafficked and unidentified patients in the EHR shadows: Shining a light by sharing the data. *PLoS One.* 2019;14(3):e0213766. <https://pubmed.ncbi.nlm.nih.gov/30870468/>. 10.1371/journal.pone.0213766; PMID 30870468; PMC6417704.

55. Basavatia A, Fret J, Lukaj A, et al. Right care for the right patient each and every time. *Cureus.* 2016;8(2):e492. <https://pubmed.ncbi.nlm.nih.gov/27014526/>. 10.7759/cureus.492; PMID 27014526; PMC4792635.

56. Landman A, Teich JM, Pruitt P, et al. The Boston Marathon bombings mass casualty incident: One emergency department’s information systems challenges and opportunities. *Ann Emerg Med.* 2015;66(1):51-59. <https://pubmed.ncbi.nlm.nih.gov/24997562/>. 10.1016/j.annemergmed.2014.06.009; PMID 24997562.

57. The Joint Commission. New and revised emergency management standards for ambulatory care programs. R3 report. 2023;39(July 30). <https://www.jointcommission.org/standards/r3-report/r3-report-issue-39-new-and-revised-emergency-management-standards-for-ambulatory-care-programs/> Accessed July 31, 2024.

58. Larsen E, Fong A, Wernz C, Ratwani RM. Implications of electronic health record downtime: An analysis of patient safety event reports. *J Am Med Inform Assoc.* 2018;25(2):187-191. <https://pubmed.ncbi.nlm.nih.gov/28575417/>. 10.1093/jamia/ocx057; PMID 28575417; PMC7647128.

59. Cohen R, Ning S, Yan MTS, Callum J. Transfusion safety: The nature and outcomes of errors in patient registration. *Transfus Med Rev.* 2019;33(2):78-83. <https://pubmed.ncbi.nlm.nih.gov/30626535/>. 10.1016/j.tmrv.2018.11.004; PMID 30626535.

60. Adelman J, Aschner J, Schechter C, et al. Use of temporary names for newborns and associated risks. *Pediatrics.* 2015;136(2):327-333. <https://pubmed.ncbi.nlm.nih.gov/26169429/>. 10.1542/peds.2015-0007; PMID 26169429.

61. Pfeifer E, Lozovatsky M, Abraham J, Kannampallil T. Effect of an alternative newborn naming strategy on wrong-patient errors: A quasi-experimental study. *Appl Clin Inform.* 2020;11(2):235-241. <https://pubmed.ncbi.nlm.nih.gov/32236916/>. 10.1055/s-0040-1705175; PMID 32236916; PMC7112998.

62. Besagar S, Robles PL, Rojas C, Applebaum JR, Adelman JS, Goffman D. Acceptability of using newborns' given names at birth: Survey in postpartum and antepartum units. *Obstet Gynecol.* 2020;135:156S-157S.

63. Janowak CF, Janowak LM. Misidentifying the unidentified – John Doe and the EHR. 2017. <https://psnet.ahrq.gov/web-mm/misidentifying-unidentified-john-doe-and-ehr>. Accessed August 1, 2024.

64. Brooks AJ, Macnab C, Boffard K. AKA unknown male Foxtrot 23/4: Alias assignment for unidentified emergency room patients. *J Accid Emerg Med.* 1999;16(3):171-173. <https://pubmed.ncbi.nlm.nih.gov/10353040/>. PMID 10353040; PMC1343326.

65. Blank-Reid CA, Kaplan LJ. A system for working with unidentified trauma patients. *Int J Trauma Nurs.* 1996;2(4):108-110. <https://pubmed.ncbi.nlm.nih.gov/9079339/>. 10.1016/S1075-4210(96)80071-X; PMID 9079339.

66. Robinson G, Fortune JB, Wachtel TL, Frank HA, Long WB. A system of alias assignment for unidentified patients requiring emergency hospital admission. *J Trauma Acute Care Surg.* 1985;25(4):333. <https://pubmed.ncbi.nlm.nih.gov/3989892/>. PMID 3989892.

67. Kaufman RM, Dinh A, Cohn CS, et al. Electronic patient identification for sample labeling reduces wrong blood in tube errors. *Transfusion.* 2019;59(3):972-980. <https://pubmed.ncbi.nlm.nih.gov/30549289/>. 10.1111/trf.15102; PMID 30549289.

68. Nayeri ND, Nadali J, Divani A, Hatefimoadab N. Ways to enhance blood transfusion safety: A systematic review. *Florence Nightingale J Nurs.* 2022;30(3):288-300. <https://pubmed.ncbi.nlm.nih.gov/36106812/>. 10.5152/fnjn.2022.21214; PMID 36106812; PMC9623141.

69. Hutton K, Ding Q, Wellman G. The effects of bar-coding technology on medication errors: A systematic literature review. *J Patient Saf.* 2021;17(3):e192-e206. <https://pubmed.ncbi.nlm.nih.gov/28234729/>. 10.1097/pts.0000000000000366; PMID 28234729.

70. Steitz BD, Li G, Wright A, Dunworth B, Freundlich RE, Wanderer JP. Non-interruptive clinical decision support to improve perioperative electronic positive patient identification. *J Med Syst.* 2022;46(3):15. <https://pubmed.ncbi.nlm.nih.gov/35079867/>. 10.1007/s10916-022-01801-7; PMID 35079867; PMC8862728.

71. Henneman PL, Marquard JL, Fisher DL, et al. Bar-code verification: Reducing but not eliminating medication errors. *J Nurs Adm.* 2012;42(12):562-566. <https://pubmed.ncbi.nlm.nih.gov/23151928/>. 10.1097/NNA.0b013e318274b545; PMID 23151928.

72. Chou SS, Chen YJ, Shen YT, Yen HF, Kuo SC. Implementation and effectiveness of a bar code-based transfusion management system for transfusion safety in a tertiary hospital: Retrospective quality improvement study. *JMIR Med Inform.* 2019;7(3):e14192. <https://pubmed.ncbi.nlm.nih.gov/31452517/>. 10.2196/14192; PMID 31452517; PMC6732972.

73. Al-Eshaq DH, Bradley RT, McBride ERA, Ford JC. Patient and specimen identification in a tertiary care pediatric hospital: Barcodes do not scan themselves. *Transfusion.* 2023;63(7):1310-1317. <https://pubmed.ncbi.nlm.nih.gov/37226989/>. 10.1111/trf.17399; PMID 37226989.

74. San TH, Lin SKS, Fai CM. Factors affecting registered nurses' use of medication administration technology in acute care settings: A systematic review. *JBI Evidence Synthesis.* 2012;10(8):471. <https://pubmed.ncbi.nlm.nih.gov/27820547/>. 10.11124/jbisrir-2012-55; PMID 27820547.

75. Barboi C, Dixon BE, McFarlane TD, Grannis SJ. *Chapter 12 - Client Registries: Identifying and linking patients.* Academic Press; 2023.

76. Rudin RS, Hillestad R, Ridgely MS, Qureshi N, Davis JS, II, Fischer SH. Defining and evaluating patient-empowered approaches to improving record matching. 2018. <https://www.rand.org/pubs/research_reports/RR2275.html>. Accessed August 1, 2024.

77. Khan LM, Slaughter RK, Bedyoa A. Policy statement of the Federal Trade Commission on biometric information and Section 5 of the Federal Trade Commission act. 2023. <https://www.ftc.gov/system/files/ftc_gov/pdf/p225402biometricpolicystatement.pdf>. Accessed July 29, 2024.

78. Jeon B, Jeong B, Jee S, et al. A facial recognition mobile app for patient safety and biometric identification: Design, development, and validation. *JMIR Mhealth Uhealth.* 2019;7(4):e11472. <https://pubmed.ncbi.nlm.nih.gov/30958275/>. 10.2196/11472; PMID 30958275; PMC6475824.

79. Wells A, Usman AB. Privacy and biometrics for smart healthcare systems: Attacks, and techniques. *Information Security Journal: A Global Perspective.* 2024;33(3):307-331. 10.1080/19393555.2023.2260818.

80. Tay KY, Pang YH, Ooi SY, Goh FL. Contactless patient authentication for registration using face recognition technology. Paper presented at: Lecture Notes in Electrical Engineering 2021.

81. Sawa M, Inoue T, Manabe S. Biometric palm vein authentication of psychiatric patients for reducing in-hospital medication errors: A pre-post observational study. *BMJ Open.* 2022;12(4):e055107. <https://pubmed.ncbi.nlm.nih.gov/35487740/>. 10.1136/bmjopen-2021-055107; PMID 35487740; PMC9058808.

82. Judson T, Haas M, Lagu T. Medical identity theft: Prevention and reconciliation initiatives at Massachusetts General Hospital. *Jt Comm J Qual Patient Saf.* 2014;40(7):291-295. <https://pubmed.ncbi.nlm.nih.gov/25130011/>. 10.1016/s1553-7250(14)40038-2; PMID 25130011.

83. The Joint Commission. People, processes, health IT and accurate patient identification. 2018. <https://www.jointcommission.org/-/media/tjc/newsletters/qs_hit_and_patient_id_9_25_18_finalpdf.pdf> Accessed July 29, 2024.

84. Salmasian H, Blanchfield BB, Joyce K, et al. Association of display of patient photographs in the electronic health record with wrong-patient order entry errors. *JAMA Netw Open.* 2020;3(11):e2019652. <https://pubmed.ncbi.nlm.nih.gov/33175173/>. 10.1001/jamanetworkopen.2020.19652; PMID 33175173; PMC7658731.

85. Rzewnicki D, Kanvinde A, Gillespie S, Orenstein E. Association of patient photographs and reduced retract-and-reorder events. *JAMIA Open.* 2024;7(3):ooae042. <https://pubmed.ncbi.nlm.nih.gov/38957593/>. 10.1093/jamiaopen/ooae042; PMID 38957593; PMC11218880.

86. Hyman D, Laire M, Redmond D, Kaplan DW. The use of patient pictures and verification screens to reduce computerized provider order entry errors. *Pediatrics.* 2012;130(1):e211-219. <https://pubmed.ncbi.nlm.nih.gov/22665415/>. 10.1542/peds.2011-2984; PMID 22665415.

87. Delgado M, Dard S, Jonsson Funk M, Carey T. Explaining the inexplicable: Irregularities in electronic health record derived data. *Pharmacoepidemiol Drug Saf.* 2020;29(S3):335. 10.1002/pds.5114.

88. Li X, Xu H, Grannis S. The data-adaptive fellegi-sunter model for probabilistic record linkage: Algorithm development and validation for incorporating missing data and field selection. *J Med Internet Res.* 2022;24(9):e33775. <https://pubmed.ncbi.nlm.nih.gov/36173664/>. 10.2196/33775; PMID 36173664; PMC9562057.

89. Shao P, Tepsick JG, Walker B, Ray HE. Improving real-world mortality data quality in oncology research: Augmenting electronic medical records with obituary, social security death index, and commercial claims data. *JCO Clin Cancer Inform.* 2023;7:e2300014. <https://pubmed.ncbi.nlm.nih.gov/37695983/>. 10.1200/cci.23.00014; PMID 37695983; PMC10569778.

90. Curtis MD, Griffith SD, Tucker M, et al. Development and validation of a high-quality composite real-world mortality endpoint. *Health Serv Res.* 2018;53(6):4460-4476. <https://pubmed.ncbi.nlm.nih.gov/29756355/>. 10.1111/1475-6773.12872; PMID 29756355; PMC6232402.

91. Conway RBN, Armistead MG, Denney MJ, Smith GS. Validating the matching of patients in the linkage of a large hospital system's EHR with state and national death databases. *Appl Clin Inform.* 2021;12(1):82-89. <https://pubmed.ncbi.nlm.nih.gov/33567463/>. 10.1055/s-0040-1722220; PMID 33567463; PMC7875675.

92. Office of the National Coordinator for Health Information Technology (ONC). Patient Identification and Matching Final Report 2014. <https://www.healthit.gov/sites/default/files/patient_identification_matching_final_report.pdf>. Accessed August 1, 2024.

93. Elkins S. Patient matching: Are we any closer to a solution? *For The Record.* 2018;30(9):18. <https://www.fortherecordmag.com/archives/1018p18.shtml>. Accessed July 23, 2024.

94. National Quality Forum. Identification and prioritization of Health IT patient safety measures. 2016. <https://www.qualityforum.org/publications/2016/02/identification_and_prioritization_of_hit_patient_safety_measures.aspx>. Accessed August 1, 2024.

95. Moscovitch B, Halamka JD, Grannis S. Better patient identification could help fight the coronavirus. *NPJ Digit Med.* 2020;3:83. <https://pubmed.ncbi.nlm.nih.gov/32529044/>. 10.1038/s41746-020-0289-4; PMID 32529044; PMC7264357.

96. Grannis SJ, Williams JL, Kasthuri S, Murray M, Xu H. Evaluation of real-world referential and probabilistic patient matching to advance patient identification strategy. *J Am Med Inform Assoc.* 2022;29(8):1409-1415. <https://pubmed.ncbi.nlm.nih.gov/35568993/>. 10.1093/jamia/ocac068; PMID 35568993; PMC9277641.

97. Arndt RZ. Fail-safe patient matching remains just out of reach. *Mod Healthc.* 2018;48(29):22. <https://psnet.ahrq.gov/issue/fail-safe-patient-id-matching-remains-just-out-reach>. Accessed July 23, 2024.

98. Gilbert R, Lafferty R, Hagger-Johnson G, et al. Guild: Guidance for information about linking data sets. *J Public Health (Oxf).* 2018;40(1):191-198. <https://pubmed.ncbi.nlm.nih.gov/28369581/>. 10.1093/pubmed/fdx037; PMID 28369581; PMC5896589.

99. SHOT Steering Group. 2019 Annual SHOT Report. *Serious Hazards of Transfusion (SHOT).* 2019. <https://hospital.blood.co.uk/the-update/2019-annual-shot-report/>.

100. Hua Y, Wang L, Nguyen V, et al. A deep learning approach for transgender and gender diverse patient identification in electronic health records. *J Biomed Inform.* 2023;147:104507. <https://pubmed.ncbi.nlm.nih.gov/37778672/>. 10.1016/j.jbi.2023.104507; PMID 37778672; PMC10687838.

101. Ross MK, Sanz J, Tep B, Follett R, Soohoo SL, Bell DS. Accuracy of an electronic health record patient linkage module evaluated between neighboring academic health care centers. *Appl Clin Inform.* 2020;11(5):725-732. <https://pubmed.ncbi.nlm.nih.gov/33147645/>. 10.1055/s-0040-1718374; PMID 33147645; PMC7641664.

102. Nagels J, Wu S, Gorokhova V. Deterministic vs. Probabilistic: Best practices for patient matching based on a comparison of two implementations. *J Digit Imaging.* 2019;32(6):919-924. <https://pubmed.ncbi.nlm.nih.gov/31292769/>. 10.1007/s10278-019-00253-9; PMID 31292769; PMC6841798.
